# A Synergistic Effect of Phthalimide-Substituted Sulfanyl Porphyrazines and Carbon Nanotubes to Improve the Electrocatalytic Detection of Hydrogen Peroxide

**DOI:** 10.3390/molecules27144409

**Published:** 2022-07-09

**Authors:** Michal Falkowski, Amanda Leda, Tomasz Rebis, Jaroslaw Piskorz, Lukasz Popenda, Mina Hassani, Dariusz T. Mlynarczyk, Michal P. Marszall, Grzegorz Milczarek

**Affiliations:** 1 Department of Medicinal Chemistry, Collegium Medicum in Bydgoszcz, Faculty of Pharmacy, Nicolaus Copernicus University in Torun, Dr. A. Jurasza 2, 85-089 Bydgoszcz, Poland; mina.hassani2022@gmail.com (M.H.); mmars@cm.umk.pl (M.P.M.); 2 Institute of Chemistry and Technical Electrochemistry, Poznan University of Technology, Berdychowo 4, 60-965 Poznan, Poland; amanda.leda@doctorate.put.poznan.pl (A.L.); grzegorz.milczarek@put.poznan.pl (G.M.); 3 Chair and Department of Inorganic and Analytical Chemistry, Poznan University of Medical Sciences, Rokietnicka 3, 60-806 Poznan, Poland; piskorzj@ump.edu.pl; 4 NanoBioMedical Centre, Adam Mickiewicz University in Poznan, Wszechnicy Piastowskiej 3, 61-614 Poznan, Poland; lpopenda@gmail.com; 5 Chair and Department of Chemical Technology of Drugs, Poznan University of Medical Sciences, Grunwaldzka 6, 60-780 Poznan, Poland; mlynarczykd@ump.edu.pl

**Keywords:** electrocatalysis, hydrogen peroxide, porphyrazine, voltammetry, carbon nanotubes

## Abstract

A sulfanyl porphyrazine derivative with peripheral phthalimide moieties was metallated with cobalt(II) and iron(II) metal ions. The purity of the macrocycles was confirmed by HPLC, and subsequently, compounds were characterized using various analytical methods (ES-TOF, MALDI-TOF, UV–VIS, and NMR spectroscopy). To obtain hybrid electroactive electrode materials, novel porphyrazines were combined with multiwalled carbon nanotubes. The electrocatalytic effect derived from cobalt(II) and iron(II) cations was evaluated. As a result, a significant decrease in the overpotential was observed compared with that obtained with bare glassy carbon (GC) or glassy carbon electrode/carbon nanotubes (GC/MWCNTs), which allowed for sensitive determination of hydrogen peroxide in neutral conditions (pH 7.4). The prepared sensor enables a linear response to H_2_O_2_ concentrations of 1–90 µM. A low detection limit of 0.18 μM and a high sensitivity of 640 μA mM^−1^ cm^−2^ were obtained. These results indicate that the obtained sensors could potentially be applied in biomedical and environmental fields.

## 1. Introduction

Hydrogen peroxide is a significant molecule widely used in biomedicine and the food and environmental industries [1]. Moreover, it is a strong chemical oxidant and is an important by-product of many biochemical reactions of enzymes from the oxidase family. Increased levels of hydrogen peroxide can potentially cause serious disorders/diseases, such as cancer, cardiovascular disease, and Alzheimer’s [2,3]. Hence, developing efficient and sensitive H_2_O_2_ sensors is required for human health and industrial process monitoring. An electrochemical method is the perfect tool for monitoring H_2_O_2_ due to the low cost of equipment, simplicity, and potential of miniaturizing the final sensing devices [4,5]. However, the detection of hydrogen peroxide on conventional electrodes requires high overpotentials, which might trigger interference from coexisting substances. To overcome such a drawback, various modified electrodes have been extensively constructed by the immobilization of efficient and selective catalysts, such as metal nanoparticles [6], metal oxides [7], metal hexacyanoferrates [8], and metal porphyrinoids (phthalocyanine, porphyrin, porphyrazine) [9,10,11]. The surface of the electrode may be also modified to produce an electrocatalyst-grafted electrode surface or the surface of the electrode may express electrocatalytic behavior itself [12,13]. The electrocatalyst takes part in the electrochemical processes and aids in the electron transfer between the electrode and the reactants in the analyte, and it may facilitate chemical transformation. Furthermore, by utilizing different electrode materials, the electrocatalysis offers the possibility of generating different electrode kinetics [14,15,16]. Among these electrocatalysts, porphyrinoids have attracted much attention in recent years. The desired electrocatalytic properties are dependent on the central metal ion (e.g., Co, Fe, Mn, Ni) as well as on substituents attached to the macrocyclic rings [17]. The application of functional macrocycles can allow the monitoring of H_2_O_2_ at lower potentials compared with ordinary electrodes.

Porphyrazines (Pzs) are synthetic tetrapyrrolic macrocycles, aza analogs of naturally occurring porphyrins. The highly conjugated porphyrazine ring grants the molecule unique properties, including optical, electrical, and photochemical, rendering it potentially useful in fields such as medicine, sensors, or photocatalysis [18,19]. The sole unmodified Pz ring is highly nonpolar and prone to π–π stacking, which hampers its broad use. Thus, Pzs may be modified in two main approaches—by changing the element complexed in the coordinating center, usually a metal cation, or by peripheral substitution at the β-positions. In this way, the physicochemical properties of the macrocycle may be tailored to a specific application. Among Pzs, sulfanyl Pzs have gained attention as they were found to express good solubility and have interesting biological [20,21], photocatalytic [22,23], electronic [24,25], and optical properties [26]. Sulfanyl Pzs bearing nitrophenoxy [27,28] and isophthaloxyalkyl substituents [29,30] recently synthesized in our group revealed a plethora of interesting photochemical and electrochemical properties. Additionally, phthalimide moieties were reported to strongly influence the macrocycles they were attached to. When phthalimide rings are fused to the macrocycle, they lower the LUMO energy of the macrocycle, which was shown by Cai et al. for naphthalocyanines [31]. Rodriguez et al., on the other hand, reported the properties of topical formulations of peripherally octasubstituted phthalocyanines, one of which was tetrasubstituted with *N*-alkylphthalimides [32].

Multiwalled carbon nanotubes (MWCNTs) were found to be outstanding electrode materials due to their high porosity, good electrical conductivity, and high chemical stability [33]. The surface of MWCNTs can easily be modified by strong noncovalent π–π stacking interactions between MWCNT and hydrophobic porphyrinoid molecules [17]. Such an approach improved the electron transfer from the electrode surface to the redox-active complexes [34]. Recently, there has been a growing interest in studying the application of macrocyclic compounds integrated with carbon nanostructures for the construction of hydrogen peroxide sensors. This is due to their attractive physicochemical properties and high catalytic activity. For instance, Hosu et al. examined that the electrocatalytic activity of cobalt(II) phthalocyanine tetracarboxylic acid (CoPc–COOH) loaded reduced graphene oxide (rGO) films to detect peroxynitrite and hydrogen peroxide [35]. In other works, Wang et al. applied cobalt(II) phthalocyanine nanorods, which were noncovalently absorbed onto graphene (Gr) through a one-step microwave-radiation-assisted synthesis, for hydrogen peroxide and glucose sensing [36]. The covalent immobilization of cobalt(II) phthalocyanine on phenylamine functionalized single-walled carbon nanotubes was reported by Mashazi et al. for the efficient sensing of H_2_O_2_ [37]. One of our previous works presented the synthesis of magnesium sulfanyl porphyrazine derivatives with peripheral hyperbranched groups and their deposition on the surface of multiwalled carbon nanotubes. The resulting hybrid electrodes with dendrimeric porphyrazines were utilized for the electrocatalytic determination of hydrogen peroxide concentration [38].

In this work, we report the synthesis and properties of a novel sulfanyl porphyrazine derivative with peripheral phthalimide moieties that was metallated with electrochemically active cobalt(II) and iron(II) cations. The voltammetric data in organic electrolytes clearly indicated defined redox features corresponding to one-electron reactions of the π-conjugated porphyrazine ring, metal center, and phthalimide substituents in the periphery. The novel porphyrazines were combined with multiwalled carbon nanotubes to obtain hybrid electroactive electrode materials. The electrochemical behavior of the immobilized phthalimide porphyrazines was consistent with the reduction mechanism involving ring- and metal-based processes together with reversible phthalimide transitions. The porphyrazine/multiwalled carbon nanotube hybrid electrodes were utilized for the electrocatalytic determination of hydrogen peroxide concentration. The electrocatalytic effect derived from the presence of cobalt(II) and iron(II) cations was evaluated. As a result, a significant decrease in the overpotential was observed compared with that obtained using a bare glassy carbon (GC) or glassy carbon electrode/carbon nanotubes (GC/MWCNTs), which allowed for the sensitive determination of hydrogen peroxide concentration in neutral conditions (pH 7.4).

## 2. Results and Discussion

### 2.1. Synthesis and Characterization

**Scheme 1:**
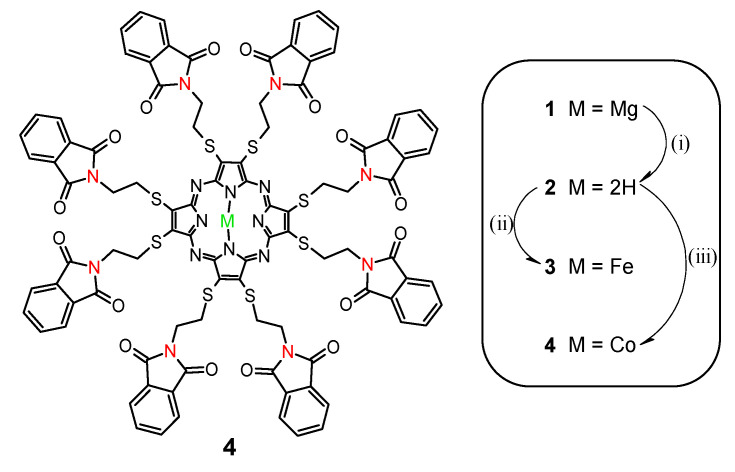
Synthesis of compounds **3-4**. Reagents and conditions: (**i**) trifluoroacetic acid, room temperature, 30 min; (**ii**) FeBr_2_, DMF, 75 °C, 24 h; (**iii**) CoCl_2_ × 6H_2_O, DMF, 75 °C, 24 h.

A magnesium(II) porphyrazine containing phthalimide substituents (**1**) and its free-base analog (**2**) were synthesized using a three-step published procedure [39]. **Pz2** was metallated in DMF with iron(II) bromide and cobalt(II) chloride hexahydrate to give iron(II) sulfanyl porphyrazine **3** and cobalt(II) sulfanyl porphyrazine **4**, respectively (Scheme 1). All compounds were purified using flash column chromatography and characterized by mass spectrometry and UV–VIS spectroscopy. NMR experiments were carried out to unambiguously identify the isolated **Pz3**. The ^1^H and ^13^C NMR resonances were assigned using a combination of 1D (^1^H, ^13^C) and 2D (^1^H–^1^H COSY, ^1^H–^13^C HSQC, and ^1^H–^13^C HMBC) experiments. A detailed analysis of the NMR spectra is presented in the Supplementary Information. In the case of **Pz4**, such characterization was not performed due to the paramagnetic nature of Co(II) [40]. Moreover, HPLC analyses of macrocyclic compounds performed in three different solvent systems confirmed the purity of new macrocycles at a level of around 100%, detecting at 380 and 670 nm (see Supplementary Information).

**Figure 1:**
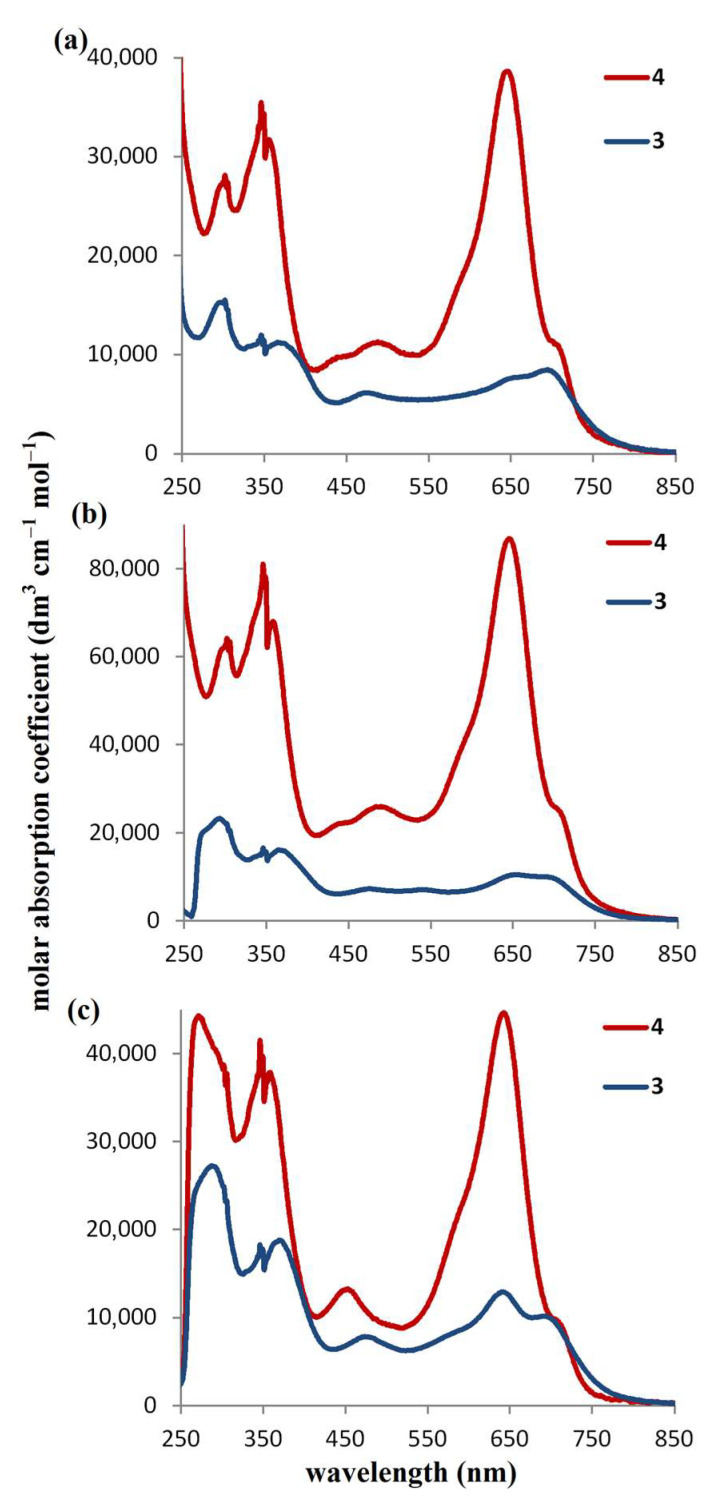
UV–VIS absorption spectra of Pzs **3** and **4** in: (**a**) dichloromethane (DCM), (**b**) *N*,*N*-dimethylformamide (DMF), and (**c**) dimethylsulfoxide (DMSO).

The absorption properties of porphyrazines **3** and **4** were determined by UV–VIS measurements in organic solvents, such as dichloromethane, *N*,*N*-dimethylformamide, and dimethyl sulfoxide (Figure 1). The change of metal cation in the macrocyclic core significantly affected the absorption bands’ position and intensity. The short wavelength Soret band showed a maxima between 365 and 370 nm for **Pz3** and in the range of 356–358 nm in the case of **Pz4**. The long-wavelength Q-band of iron(II) complex **3** was broad, had low intensity, and was divided into two incompletely developed sub-bands with maxima in various solvents at 641–655 and 691–695 nm. The logarithms of the molar absorption coefficients (log ε) were in the range 3.89–4.11. Cobalt(II) porphyrazine **4** showed a more intense, sharp Q-band with a maxima at 643–646 nm and log ε values of 4.59–4.94 (Table S2 in the Supplementary Information). It is worth noting that the introduction of the cobalt(II) cation in the porphyrazine core produced a significant Q-band hypsochromic shift of about 25–30 nm compared with the previously elaborated magnesium(II) and zinc(II) analogues [39].

### 2.2. Electrochemical Characterization of Porphyrazines in Organic Electrolyte

**Figure 2:**
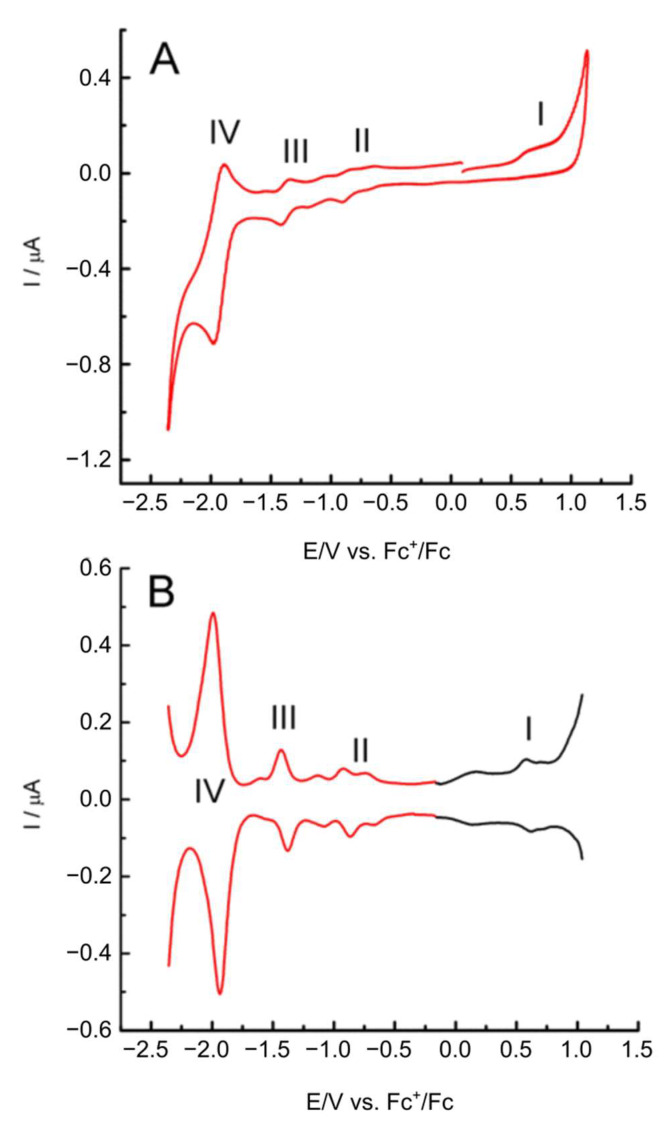
Cyclic voltammogram (**A**) and differential pulse voltammograms (**B**) for iron(II) containing porphyrazine **3**, recorded in 0.1 M DCM/TBAP.

**Figure 3:**
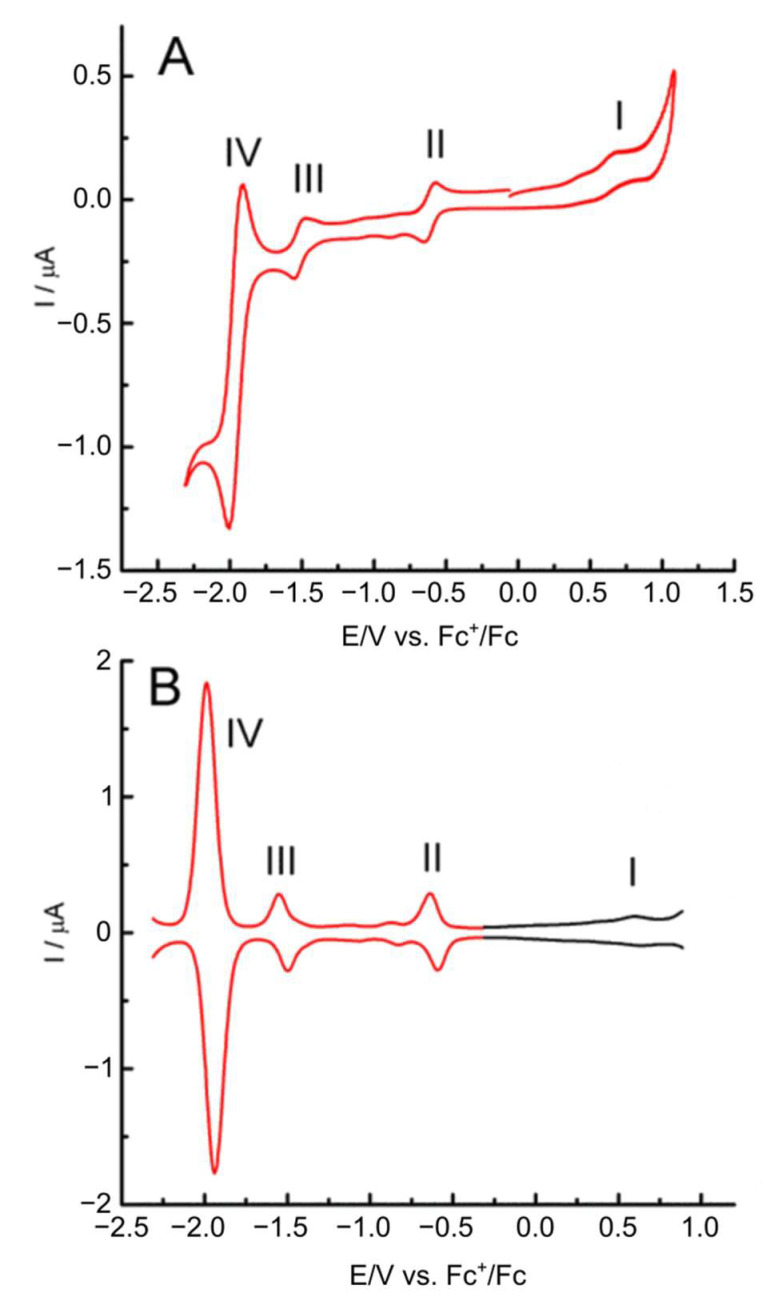
Cyclic voltammogram (**A**) and differential pulse voltammograms (**B**) for cobalt(II) containing porphyrazine **Pz4**, recorded in 0.1 M DCM/TBAP.

The cyclic voltammetry (CV) and differential pulse voltammetry (DPV) measurements of **Pz3** and **Pz4** in 0.1 M DCM/TBAP are shown in Figure 2 and Figure 3, respectively. In addition, control measurements for **Pz1** and **Pz2** are presented in Figures S4 and S5 in the Supplementary Information. All porphyrazines exhibited four well-defined redox couples. The values of E_1/2_ obtained for the porphyrazines are collected in Table 1. According to the literature data, the redox couples of magnesium(II)-based **Pz1** and nonmetallated complex **Pz2** can be assigned to ring-based one-electron processes [41,42,43,44]. In the case of complexes **Pz3** and **Pz4**, the couples marked as I can be ascribed to one-electron oxidation/reduction of π-conjugated rings [45]. However, when the potential of couple II is analyzed, a difference in the redox response of **Pz1** and **Pz2** compared with that of **Pz3** and **Pz4** can be observed. The redox peaks of couple II for **Pz3** and **Pz4** are significantly shifted towards more positive potentials compared with controls **Pz1** and **Pz2**. This suggests that couple II corresponds to metal-based redox transitions. Metal ions, such as iron(II) or cobalt(II), in the macrocyclic center of various porphyrinoids were reported to reveal electroactivity in organic electrolytes [46,47]. 

**Scheme 2:**
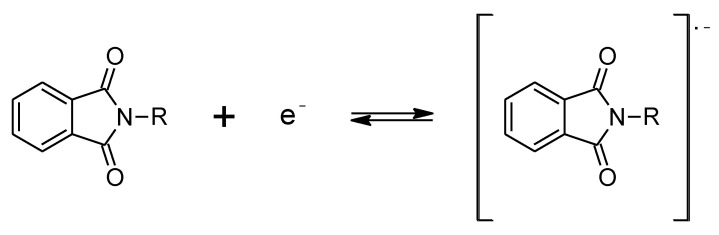
Mechanism of the reversible reduction/oxidation of phthalimide substituents in the studied macrocycles (based on [48]).

The presence of phthalimide substituents significantly affected the electrochemical response of the studied porphyrazines. A well-defined and very intense redox couple appeared at ca. −2 V for all the studied compounds. According to previous studies concerning the electrochemical behavior of phthalimide in organic electrolyte media, this couple was assigned to the electrochemical one-electron reduction/oxidation of the eight phthalimide substituents to their corresponding anion radicals (Scheme 2) [48].

### 2.3. Electrochemical Study of Porphyrazines Deposited on MWCNT

**Figure 4:**
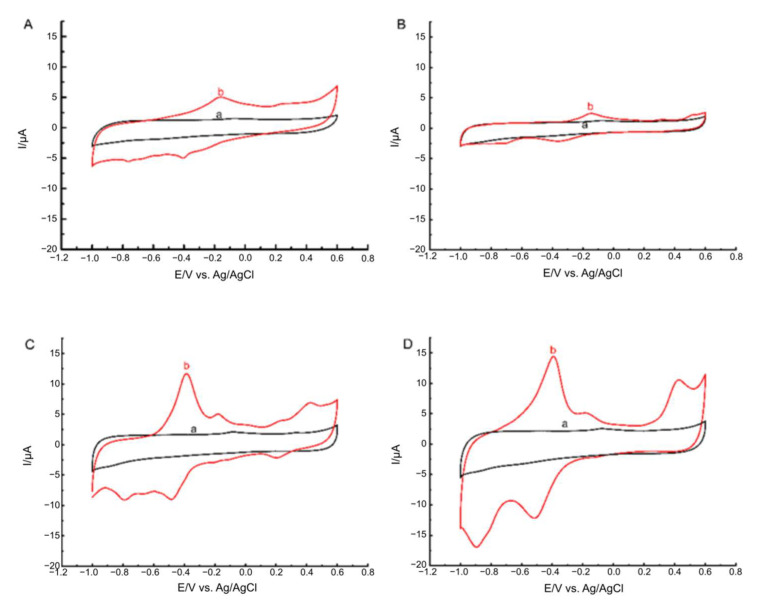
CVs of hybrid materials recorded in PB (pH = 7.4). Scan rate of 50 mV s^−1^. In all the voltammograms, the GC/MWCNT is marked by a black line (a), and the red line (b) corresponds to (**A**) GC/MWCNT/**Pz1**, (**B**) GC/MWCNT/**Pz2**, (**C**) GC/MWCNT/**Pz3**, and (**D**) GC/MWCNT/**Pz4**.

The synthesized porphyrazines were separately immobilized on the surface of MWCNT, and the voltammetric responses of the as-designed hybrid electrodes are presented in Figure 4. The voltammetric measurements were performed in an aqueous buffered (pH 7.4) electrolyte to ascertain the electrochemical activity. The CV scans recorded between −0.8 and 0.6 V vs. Ag/AgCl for GC/MWCNT present typical capacitive characteristics without redox peaks. After deposition of porphyrazines, pronounced features appeared at a negative potential range for all the functionalized electrodes. These redox couples correspond to the electrochemical transition of phthalimide substituents. The additional peaks appearing in the range of 0.2 to 0.4 can be assigned to an iron-based Fe^2+^/Fe^3+^ transition (Figure 4C) and Co^+^/Co^2+^ (Figure 4D) [49,50].

The electroactive metal loading can be roughly estimated based on the charge corresponding to the voltammetric peak. After the integration of peaks corresponding to iron(II) and cobalt(II), we estimated the metal loadings of 9.19 and 14.5 ng for iron(II) and cobalt(II), respectively.

**Figure 5:**
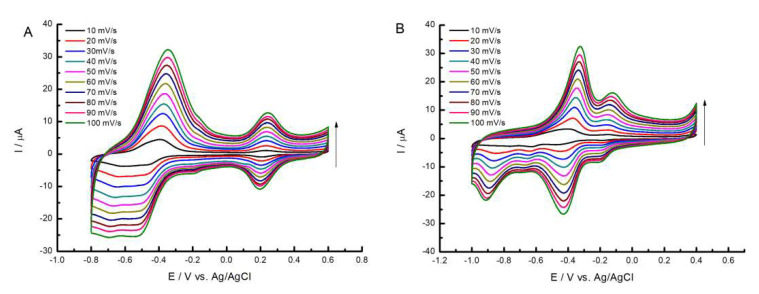
CVs of hybrid electrodes recorded in PB (pH = 7.4) at various scan rates from 10 to 100 mV s^−1^. (**A**) GC/MWCNT/**Pz3**, (**B**) GC/MWCNT/**Pz4**.

In order to study the electron transfer kinetics at the surface of both GC/MWCNT/**Pz3** and GC/MWCNT/**Pz4**, the CV measurements were conducted at various scan rates from 10 to 100 mV s^−1^ (Figure 5). The peak currents increased linearly with the scan rate, which indicates surface-controlled redox processes. The π–π stacking between the highly delocalized π-bonding network of MWCNT and the conjugated ring of porphyrazines enables surface-confined redox characteristics. Such an experiment can also prove that **Pz3** and **Pz4** do not diffuse out from the electrode surface.

### 2.4. The Influence of Hydrogen Peroxide on the Modification of the Surface of the GC Electrode

**Figure 6:**
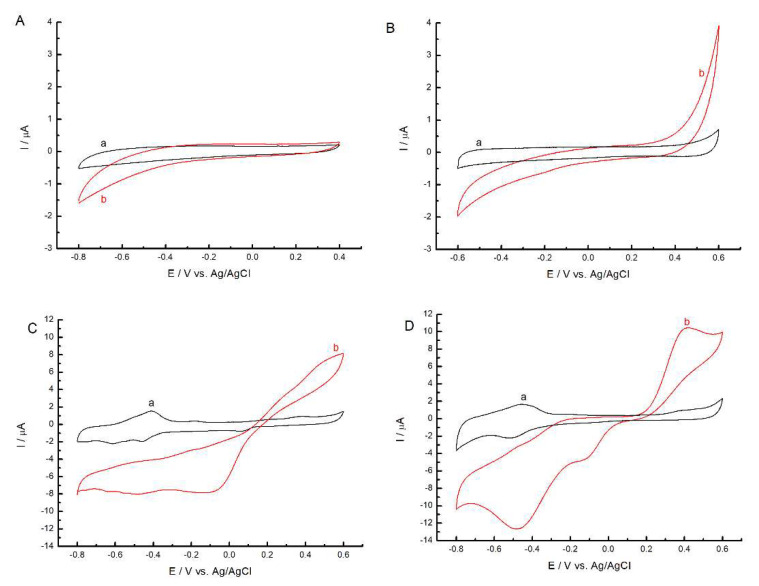
Cyclic voltammograms of (**A**) GC, (**B**) GC/MWCNT, (**C**) GC/MWCNT/**Pz3**, and (**D** )GC/MWCNT/**Pz4** recorded in PB buffer before (a) and after 2 mM H_2_O_2_ addition (b). Scan rate of 10 mV s^−1^.

Electrochemical oxidation or reduction of hydrogen peroxide is a challenging task as this molecule is a coproduct of enzymatic reactions. Hence, effective hydrogen peroxide catalysts are necessary for the development of enzymatic biosensors [51]. Porphyrinoids bearing transition metal ion centers are recognized to be promising candidates for the electrochemical catalysis of H_2_O_2_. The electrocatalytic performance of cobalt(II)- and iron(II)-based porphyrazines (**Pz3** and **Pz4**, respectively) was studied in the presence of hydrogen peroxide (Figure 6). The performance of the constructed hybrid electrodes was compared with those of bare GC and GC/MWCNT electrodes. As shown in Figure 6A, H_2_O_2_ can be reduced on bare GC at a highly negative overpotential, and the observed reductive current is relatively small. A slight improvement in H_2_O_2_ redox behavior can be observed when a GC/MWCNT electrode was used (Figure 6B). In such a case, both cathodic and anodic current waves appeared; however, the voltammetric curve revealed electrochemical irreversibility. 

Unlike GC and GC/MWCNT, the hybrid electrodes GC/MWCNT/**Pz3** and GC/MWCNT/**Pz4** revealed significant electrocatalytic activity. It is displayed in Figure 6C (curve b) that after the addition of 2 mM H_2_O_2_, a strong enhancement of the redox peaks occurs. Both reduction and oxidation of H_2_O_2_ are seen at GC/MWCNT/**Pz3** with a minor overpotential and high current. These results suggest that GC/MWCNT/**Pz3** is an appropriate electrode for the electrocatalysis of hydrogen peroxide. A well-defined anodic peak current from hydrogen peroxide is also observed for GC/MWCNT/**Pz4** (Figure 6D), suggesting that GC/MWCNT/**Pz4** has good electrocatalytic performance. However, in this case, the reduction of hydrogen peroxide is limited, and the lack of a redox signal is observed in the cathodic range. Because of that, GC/MWCNT/**Pz4** can only be applied as a catalyst for the oxidation of H_2_O_2_.

**Figure 7:**
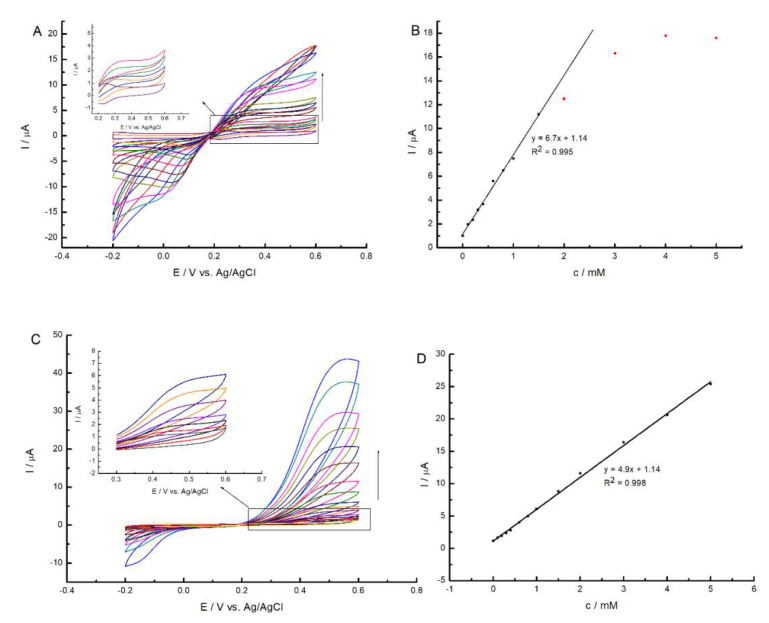
Cyclic voltammograms recorded with the increasing concentrations of H_2_O_2_ for (**A**) GC/MWCNT/**Pz3** and (**C**) GC/MWCNT/**Pz4** electrodes. (**B**) and (**D**) show the corresponding I = f (c) graphs for (**B**) GC/MWCNT/**Pz3** and (**D**) GC/MWCNT/**Pz4** electrodes. Insets in (**A**) and (**C**) are magnifications of the voltammograms in the marked range.

Cyclic voltammograms recorded for GC/MWCNT/**Pz3** and GC/MWCNT/**Pz4** in the presence of increasing concentrations of hydrogen peroxide are presented in Figure 7. The appearance and increase of well-defined peaks can be observed after consecutive additions of H_2_O_2_. The anodic currents increased linearly from 0.2 to 1.5 mM and from 0.2 to 5 mM in the case of GC/MWCNT/**Pz3** and GC/MWCNT/**Pz4**, respectively. The collected data indicate the possibility of H_2_O_2_ measurements in a broad concentration range.

### 2.5. Chronoamperometric Measurements of GC/MWCNT/Pz3- and GC/MWCNT/Pz4-Modified Electrode in the Presence of H_2_O_2_

**Figure 8:**
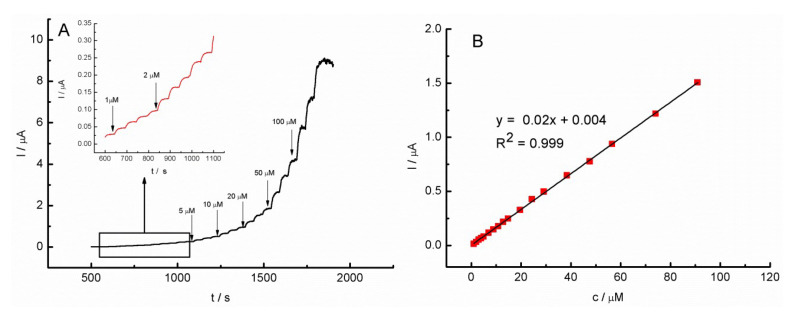
(**A**) Chronoamperometric analysis of H_2_O_2_ determination at the GC/MWCNT/**Pz3** electrode at stirring conditions. Operation potential of +0.4 V. (**B**) Dependence of the amperometric current response on the concentration of H_2_O_2_.

**Figure 9:**
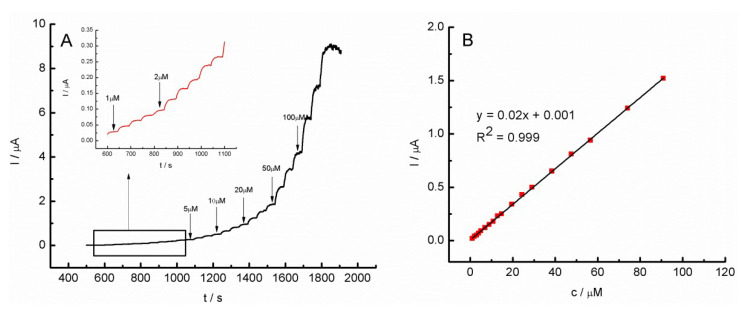
(**A**) Chronoamperometric analysis of H_2_O_2_ determination at the GC/MWCNT/**Pz4** electrode in stirring conditions. Operation potential of +0.4 V. (**B**) Dependence of the amperometric current response on the concentration of H_2_O_2_.

Additionally, chronoamperometric measurements at GC/MWCNT/**Pz3** and GC/MWCNT/**Pz4** were performed under stirring conditions (Figure 8 and Figure 9, respectively). The applied anodic potential of 0.4 V was sufficient to drive the oxidation of H_2_O_2_ at the studied electrodes. Figure 8 and Figure 9 display the amperometric signal increase after adding small amounts of hydrogen peroxide. In the case of GC/MWCNT/**Pz3,** linearity was observed within 1 to 90 µM of H_2_O_2_. Similarly, GC/MWCNT/**Pz4** presented linearity in the range of 1 to 90 µM. The estimated limits of detection (LODs) were 0.2 and 0.18 µM for GC/MWCNT/**Pz3** and GC/MWCNT/**Pz4**, respectively, taking into account a signal-to-noise ratio of 3.

A comparison of the performance of the studied electrodes for H_2_O_2_ electroanalysis with that of electrodes previously reported in the literature is shown in Table 2. The linear ranges obtained here are rather narrow; however, they are in good accordance with literature data. The main advantage of our electrodes are the relatively low LOD values. Moreover, the sensitivities of GC/MWCNT/**Pz3** and GC/MWCNT/**Pz4** are relatively high and comparable to the highest reported values.

The electrode response was carried out in the presence of common interferents that can be found in various biological systems (glucose, fructose, sodium chloride, uric acid, caffeine). To evaluate the selectivity of the GC/MWCNT/**Pz3** and GC/MWCNT/**Pz4** electrodes, the amperometric response of the electrodes was measured under stirring conditions at 0.4 V, as shown in Figure S6 (in the Supplementary Information). The addition of hydrogen peroxide resulted in a rapid current response, while the addition of most of the interfering compounds produced no current responses. However, strong interferences were recorded for uric acid. 

## 3. Materials and Methods

### 3.1. General Procedures 

All reactions were conducted in oven-dried glassware under argon atmosphere. All solvents were rotary-evaporated at or below 60 °C under reduced pressure. The reported reaction temperatures refer to Radleys^®^ Heat-On display temperatures. Dry flash column chromatography was carried out on Merck silica gel 60, particle size of 40–63 μm. Thin-layer chromatography (TLC) was performed on silica gel Merck Kieselgel 60 F254 plates and visualized with UV light (λ_max_ = 254 or 365 nm). UV–VIS spectra were recorded on a Hitachi UV–VIS U-1900 spectrophotometer. The NMR spectra were acquired at 298 K on an Agilent DD2 800 spectrometer operating at resonance frequencies of 799.903 and 201.146 MHz for ^1^H and ^13^C, respectively. Chemical shifts (δ) are quoted in parts per million (ppm) and are referenced to a residual solvent peak. Coupling constants (*J*) are quoted in hertz (Hz). The abbreviations t and m refer to triplet and multiplet, respectively. ^1^H and ^13^C resonances were unambiguously assigned based on ^1^H, ^13^C, ^1^H–^1^H COSY, ^1^H–^13^C HSQC, and ^1^H–^13^C HMBC spectra. Mass spectra (MALDI-TOF, HRMS) were carried out by the Wielkopolska Center for Advanced Technologies at the Adam Mickiewicz University in Poznan and at the European Center for Bioinformatics and Genomics in Poznan. All solvents and reagents were of reagent-grade quality, purchased from commercial suppliers, and used without further purification unless otherwise stated.

### 3.2. Synthetic Procedures and Characterization

**2,3,7,8,12,13,17,18-Octakis[(*N*-ethylphthalimide)thio]porphyrazinato magnesium(II)** (**Pz1**) and **2,3,7,8,12,13,17,18-octakis[(*N*-ethylphthalimide)thio]porphyrazine** (**Pz2**) were synthesized using a previously published procedure [39].

**2,3,7,8,12,13,17,18-Octakis[(*N*-ethylphthalimide)thio]porphyrazinato iron(II)** (**Pz3**)

Compound **Pz2** (120 mg, 0.061 mmol) and iron(II) bromide (132 mg, 0.610 mmol) were dissolved in anhydrous DMF (15 mL) and stirred at 75 °C for 24 h. Next, the reaction mixture was cooled to room temperature and filtered through Celite, which was subsequently washed with dichloromethane (150 mL). The combined filtrates were evaporated to dryness, and a brown residue was chromatographed in the normal phase (eluents: dichloromethane/ethanol, 100:1 to 20:1, *v*/*v*) to give compound **Pz3** as a violet film (47 mg, 38% yield). R*_f_* (dichloromethane/methanol, 20:1, *v*/*v*) 0.38. UV–VIS (dichloromethane) λ_max_ nm (log ε) 296 (4.18), 368 (4.05), 655 (3.89), 695 (3.93). ^1^H NMR (800 MHz, pyridine-*d_5_*) δ, ppm: 7.73 (m, 16H, C2, C5, ArH), 7.55 (m, 16H, C3, C4, ArH), 4.61 (t, *J* = 6 Hz, 16H, SCH_2_), 4.39 (t, *J* = 6 Hz, 16H, NCH_2_). ^13^C NMR (201 MHz, pyridine-*d_5_*) δ, ppm: 168.6 (C=O), 152.3 (C2, C4, pyrrole C), 143.2 (C2, C3, pyrrole C), 134.6 (C3, C4, ArC), 133.0 (C1, C6, ArC), 123.8 (C2, C5, ArC), 38.9 (NCH_2_), 33.5 (SCH_2_). MS (MALDI) *m*/*z*: 2009 [M+H]^+^, 2031 [M+Na]^+^, 2047 [M+K]^+^. HRMS (ESI) *m*/*z*: found 2009.1819, [M+H]^+^ C_96_H_65_FeN_16_O_16_S_8_ requires 2009.1880. HPLC purity ~100.0% (see Supplementary Information).

**2,3,7,8,12,13,17,18-Octakis[(*N*-ethylphthalimide)thio]porphyrazinato cobalt(II)** (**Pz4**) 

Compound **Pz2** (126 mg, 0.064 mmol) and cobalt(II) chloride hexahydrate (76 mg, 0.32 mmol) were dissolved in anhydrous DMF (15 mL) and stirred at 75 °C for 24 h. Next, the reaction mixture was cooled to room temperature and filtered through Celite, which was subsequently washed with dichloromethane (150 mL). The combined filtrates were evaporated to dryness, and a blue residue was chromatographed in the normal phase (dichloromethane/methanol, gradient 100:1 to 20:1, *v*/*v*) to give compound **Pz4** as a navy blue film (105 mg, 81%). R*_f_* (dichloromethane/methanol, 100:1, *v*/*v*) 0.30. UV–VIS (dichloromethane) λ_max_ nm (log ε) 297 (4.43), 356 (4.50), 645 (4.59). MS (MALDI) *m*/*z*: 2012 [M+H]^+^, 2034 [M+Na]^+^. HRMS (ESI) *m*/*z*: found 2012.1805, [M+H]^+^ C_96_H_65_CoN_16_O_16_S_8_ requires 2012.1862. HPLC purity ~100.0% (see Supplementary Information).

### 3.3. Materials and Reagents for Electrochemical Testing

Multiwalled carbon nanotubes (MWCNTs, with an average diameter of 10 nm and an average length of 1.5 µm) were delivered by Metrohm DropSens (Oviedo, Spain). Monosodium and disodium phosphates for the preparation of phosphate buffer (PB, pH 7.4) were supplied by Avantor (Gliwice, Poland). Dichloromethane (DCM, puriss. p.a., ≥99.9%), *N*,*N*-dimethylformamide (DMF, anhydrous 99.8%), tetrabutylammonium perchlorate (TBAP, ≥99.9%), and hydrogen peroxide stock solution 30 wt. % were purchased from Merck (Darmstadt, Germany). 

### 3.4. Electrochemical Methods 

A PalmSens electrochemical analyzer was used for the electrochemical testing (Eindhoven, Netherlands). A three-electrode setup was employed, with a reference electrode of Ag/AgCl (3 M KCl) and a counter electrode of platinum wire. The working electrode was a glassy carbon electrode (GC) with a diameter of 2 mm (Mineral, Poland). For organic experiments, a silver wire was used as a reference electrode (instead of Ag/AgCl). Ferrocene (Fc) was added at the end of each experiment. The electrode potentials were set versus the ferrocenium/ferrocene couple (Fc^+^/Fc).

### 3.5. Fabrication of GC/MWCNT, GC/MWCNT/Pz1, GC/MWCNT/Pz2, GC/MWCNT/Pz3, and GC/MWCNT/Pz4 Electrodes

The GC electrode was polished with an aqueous suspension of Al_2_O_3_ (Buehler, 50 nm average diameter) on a polishing cloth before each electrochemical measurement and then washed in an ultrasonic bath with 1:1 *v*/*v* water/acetone for 10 min to eliminate any contaminants. An amount of 2 μL of MWCNT (1 mg mL^−1^ suspension in DMF) was drop-cast on the cleaned GC electrode surface, and the electrode was dried in an oven (60 °C) to evaporate the solvent. Then **Pz1**, **Pz2**, **Pz3**, and **Pz4** each were dissolved in dichloromethane to give a solution of 1 mg mL^−1^. The resultant GC/MWCNT electrodes were immersed in the previously prepared solutions of either **Pz1**, **Pz2**, **Pz3**, or **Pz4**. Noncovalent immobilization of the porphyrazines was carried out by incubating the GC/MWCNT electrodes in the solutions. To perform electrochemical testing, all electrodes were placed in the desired electrolyte. Before the experiments, a glass cell containing supporting electrolytes was deoxygenated by purging with pure nitrogen gas. All electrochemical experiments were carried out at an ambient laboratory temperature (ca. 25 °C).

## 4. Conclusions

The chemical synthesis of new metallated iron(II) and cobalt(II) porphyrazines with phthalimide moieties was elaborated. All the obtained compounds were characterized using spectral techniques, and their purity was verified using HPLC. Hybrid nanomaterials based on novel porphyrazine and multiwalled carbon nanotubes were produced utilizing noncovalent π–π interactions. Interestingly, all the studied porphyrazines adsorbed on the MWCNT surface revealed well–defined redox couples, which were ascribed to phthalimide electron transfer. The application of hybrid electrodes for the electrocatalytic determination of hydrogen peroxide was evaluated. Cobalt(II) and iron(II) containing porphyrazines provided reversible and accessible metal-based redox sites, which acted as an electrocatalyst toward H_2_O_2_. The prepared sensor enabled a linear response to H_2_O_2_ concentrations of 1–90 µM. A limit of detection of 0.18 μM was obtained, revealing the high sensitivity of the GC/MWCNT/**Pz4** electrode (640 μA mM^−1^ cm^−2^). Currently, work is in progress to apply the presented electrodes as hydrogen peroxide catalysts to develop an enzymatic biosensor for glucose determination.

## Figures and Tables

**Table 1:** Comparison of E_1/2_ for the studied compounds in 0.1 M DCM/TBAP electrolyte.

Compound	E_1/2_ I/V	E_1/2_ II/V	E_1/2_ III/V	E_1/2_ I*V*/*V*
**Pz1** (metal-free)	0.90	−0.89	−1.13	−1.98
**Pz2** (Mg)	0.63	−1.14	−1.40	−1.99
**Pz3** (Fe)	0.61	−0.82/−0.62 *	−1.37	−1.96
**Pz4** (Co)	0.63	−0.62	−1.50	−1.96

* Value estimated for II/II^’^ redox couple; E^1/2^ estimated form CV measurements recorded at 50 mV s^−1^.

**Table 2:** Comparison of the analytical performance of the proposed electrodes with those of other reported hydrogen peroxide sensors.

Electrode	Sensitivity/μA mM^−1^ cm^−2^	LOD/μM	Linear Range/mM	Ref.
MWCNT/LS/NAg	252	1.17	6–486	[12]
AgNPs/GC	169	2.0	5–50	[52]
Ag-exGRc-CI/SS	115	5.0	100–8000	[53]
PANI-Ag/GCE	-	0.23	10–90	[54]
AuP/GCE	-	4	100–50,000	[55]
Pd/MCV/Nafion/GC	-	0.079	0.1–6100	[56]
GC/MWCNT/Pz3	636	0.20	1–90	This work
GC/MWCNT/Pz4	640	0.18	1–90	This work

## Data Availability

Not applicable.

## Supplementary Materials

The following supporting information can be downloaded at https://www.mdpi.com/article/10.3390/molecules27144409/s1, Figure S1: ^1^H and (^13^C) chemical shift values [ppm] of **3** and key correlations observed in NMR spectra. Figure S2: ^1^H NMR spectrum of **3** (800 MHz, DMSO-*d*_6_, 298 K). Figure S3: ^13^C NMR spectrum of **3** (201 MHz, DMSO-*d*_6_, 298 K). Figure S4: Cyclic voltammogram (**A**) and differential pulse voltammograms (**B**) for magnesium(II) porphyrazine **Pz1**. Figure S5: Cyclic voltammogram (**A**) and differential pulse voltammograms (**B**) for metal-free porphyrazine **Pz2**. Figure S6: Chronomperometric response of the GC/MWCNT/**Pz3** (**A**) and GC/MWCNT/**Pz4** (**B**). Table S1: ^1^H and ^13^C NMR data obtained for **3** including key correlations determined from ^1^H-^13^C HSQC and ^1^H-^13^C HMBC spectra. Table S2: UV–Vis absorption maxima (λ_Abs_) and logarithms of molar absorption coefficients (logε) of Pzs **3** and **4** in selected organic solvents.

## Sample Availability

Not available.

## References

[1] Xing L., Zhang W., Fu L., Lorenzo J.M., Hao Y. (2022). Fabrication and Application of Electrochemical Sensor for Analyzing Hydrogen Peroxide in Food System and Biological Samples. Food Chem..

[2] Deng Z., Zhao L., Zhou H., Xu X., Zheng W. (2022). Recent Advances in Electrochemical Analysis of Hydrogen Peroxide towards in Vivo Detection. Process Biochem..

[3] Byon C.H., Heath J.M., Chen Y. (2016). Redox Signaling in Cardiovascular Pathophysiology: A Focus on Hydrogen Peroxide and Vascular Smooth Muscle Cells. Redox Biol..

[4] Ghalkhani M., Gharagozlou M., Sohouli E., Marzi Khosrowshahi E. (2022). Preparation of an Electrochemical Sensor Based on a HKUST-1/CoFe2O4/SiO2-Modified Carbon Paste Electrode for Determination of Azaperone. Microchem. J..

[5] Ghalkhani M., Khosrowshahi E.M., Sohouli E., Eskandari K., Aghaei M., Rahimi-Nasrabadi M., Sobhani-Nasab A., Banafshe H., Kouchaki E. (2022). Electrochemical Monitoring of Carbamazepine in Biological Fluids by a Glassy Carbon Electrode Modified with CuO/ZnFe_2_O_4_/RGO Nanocomposite. Surf. Interfaces.

[6] Ghosh S., Singh P., Roy S., Bhardwaj K., Jaiswal A. (2022). Superior Peroxidase-Like Activity of Gold Nanorattles in Ultrasensitive H2O2 Sensing and Antioxidant Screening. ChemBioChem.

[7] Ullah R., Rasheed M.A., Abbas S., Rehman K., Shah A., Ullah K., Khan Y., Bibi M., Ahmad M., Ali G. (2022). Electrochemical Sensing of H_2_O_2_ Using Cobalt Oxide Modified TiO_2_ Nanotubes. Curr. Appl. Phys..

[8] Virbickas P., Kavaliauskaitė G., Valiūnienė A., Plaušinaitienė V., Rekertaitė A.I., Ramanavičius A. (2020). Cobalt Hexacyanoferrate Based Optical Sensor for Continuous Optical Sensing of Hydrogen Peroxide. Electrochim. Acta.

[9] Mounesh, Reddy K.R.V. (2020). Detection of Nanomolar Concentrations H_2_O_2_ Using Cobalt (II) Phthalocyanine Modified GCE with MWCNTs. Anal. Chem. Lett..

[10] Fagadar-Cosma E., Plesu N., Lascu A., Anghel D., Cazacu M., Ianasi C., Fagadar-Cosma G., Fratilescu I., Epuran C. (2020). Novel Platinum-Porphyrin as Sensing Compound for Efficient Fluorescent and Electrochemical Detection of H_2_O_2_. Chemosensors.

[11] Falkowski M., Rebis T., Kryjewski M., Popenda L., Lijewski S., Jurga S., Mielcarek J., Milczarek G., Goslinski T. (2017). An Enhanced Electrochemical Nanohybrid Sensing Platform Consisting of Reduced Graphene Oxide and Sulfanyl Metalloporphyrazines for Sensitive Determination of Hydrogen Peroxide and L-Cysteine. Dye. Pigment..

[12] Płócienniczak P., Rębiś T., Nowicki M., Milczarek G. (2021). A Green Approach for Hybrid Material Preparation Based on Carbon Nanotubes/Lignosulfonate Decorated with Silver Nanostructures for Electrocatalytic Sensing of H_2_O_2_. J. Electroanal. Chem..

[13] Sahoo S., Sahoo P.K., Manna S., Satpati A.K. (2020). A Novel Low Cost Nonenzymatic Hydrogen Peroxide Sensor Based on CoFe_2_O_4_/CNTs Nanocomposite Modified Electrode. J. Electroanal. Chem..

[14] Maringa A., Antunes E., Nyokong T. (2014). Electrochemical Behaviour of Gold Nanoparticles and Co Tetraaminophthalocyanine on Glassy Carbon Electrode. Electrochim. Acta.

[15] Tavakkoli H., Akhond M., Ghorbankhani G.A., Absalan G. (2020). Electrochemical Sensing of Hydrogen Peroxide Using a Glassy Carbon Electrode Modified with Multiwalled Carbon Nanotubes and Zein Nanoparticle Composites: Application to HepG2 Cancer Cell Detection. Microchim. Acta.

[16] Lin Z., Zhang Q., Pan J., Tsounis C., Esmailpour A.A., Xi S., Yang H.Y., Han Z., Yun J., Amal R. (2022). Atomic Co Decorated Free-Standing Graphene Electrode Assembly for Efficient Hydrogen Peroxide Production in Acid. Energy Environ. Sci..

[17] Zagal J.H., Griveau S., Silva J.F., Nyokong T., Bedioui F. (2010). Metallophthalocyanine-Based Molecular Materials as Catalysts for Electrochemical Reactions. Coord. Chem. Rev..

[18] Fuchter M.J., Zhong C., Zong H., Hoffman B.M., Barrett A.G.M. (2008). Porphyrazines: Designer Macrocycles by Peripheral Substituent Change. Aust. J. Chem..

[19] Rodríguez-Morgade M.S., Stuzhin P.A. (2004). The Chemistry of Porphyrazines: An Overview. J. Porphyr. Phthalocyanines.

[20] Mlynarczyk D.T., Dlugaszewska J., Falkowski M., Popenda L., Kryjewski M., Szczolko W., Jurga S., Mielcarek J., Goslinski T. (2020). Tribenzoporphyrazines with Dendrimeric Peripheral Substituents and Their Promising Photocytotoxic Activity against *Staphylococcus aureus*. J. Photochem. Photobiol. B Biol..

[21] Piskorz J., Lijewski S., Gierszewski M., Gorniak K., Sobotta L., Wicher B., Tykarska E., Düzgüneş N., Konopka K., Sikorski M. (2017). Sulfanyl Porphyrazines: Molecular Barrel-like Self-Assembly in Crystals, Optical Properties and in Vitro Photodynamic Activity towards Cancer Cells. Dye. Pigment..

[22] Yin J., Zhang Q., Yang C., Zhang B., Deng K. (2020). Highly Selective Oxidation of Glucose to Gluconic Acid and Glucaric Acid in Water Catalyzed by an Efficient Synergistic Photocatalytic System. Catal. Sci. Technol..

[23] Ge Y., Zhang Q., Yang C., Zhang B., Deng K. (2021). Efficient Visible-Light-Driven Selective Conversion of Glucose to High-Value Chemicals over Bi2WO6/Co-Thioporphyrazine Composite in Aqueous Media. Appl. Catal. A Gen..

[24] Belviso S., Cammarota F., Rossano R., Lelj F. (2016). Effect of Polyfluorination on Self-Assembling and Electronic Properties of Thioalkyl-Porphyrazines. J. Porphyr. Phthalocyanines.

[25] Koczorowski T., Szczolko W., Teubert A., Goslinski T. (2021). Sulfanyl Porphyrazines with Morpholinylethyl Periphery—Synthesis, Electrochemistry, and Photocatalytic Studies after Deposition on Titanium(IV) Oxide P25 Nanoparticles. Molecules.

[26] Porolnik W., Kasprzycka M., Teubert A., Piskorz J. (2021). Serendipitous Synthesis of Unsymmetrical Porphyrazine: Incomplete Transesterification during Macrocyclization. Inorg. Chem. Commun..

[27] Rębiś T., Falkowski M., Milczarek G., Goslinski T. (2020). Electrocatalytic NADH Sensing Using Electrodes Modified with 2-[2-(4-Nitrophenoxy)Ethoxy]Ethylthio-Substituted Porphyrazine/Single-Walled Carbon Nanotube Hybrids. ChemElectroChem.

[28] Falkowski M., Rebis T., Piskorz J., Popenda L., Jurga S., Mielcarek J., Milczarek G., Goslinski T. (2017). Multiwalled Carbon Nanotube/Sulfanyl Porphyrazine Hybrids Deposited on Glassy Carbon Electrode—Effect of Nitro Peripheral Groups on Electrochemical Properties. J. Porphyr. Phthalocyanines.

[29] Rębiś T., Falkowski M., Kryjewski M., Popenda L., Sobotta L., Jurga S., Marszall M.P., Mielcarek J., Milczarek G., Goslinski T. (2019). Single-Walled Carbon Nanotube/Sulfanyl Porphyrazine Hybrids Deposited on Glassy Carbon Electrode for Sensitive Determination of Nitrites. Dye. Pigment..

[30] Stolarska M., Glowacka-Sobotta A., Ziental D., Dlugaszewska J., Falkowski M., Mielcarek J., Goslinski T., Sobotta L. (2021). Photochemical Properties and Photocytotoxicities against Wound Bacteria of Sulfanyl Porphyrazines with Bulky Peripheral Substituents. J. Organomet. Chem..

[31] Cai C., Wang L., Hu M., Li L., Fu J., Zhao X., Zhang Y., Hu Y., Yuan Z. (2022). Solution-Processable Silicon Naphthalocyanine Tetraimides as near Infrared Electron Acceptors in Organic Solar Cells. Dye. Pigment..

[32] Rodriguez M.E., Diz V.E., Awruch J., Dicelio L.E. (2010). Photophysics of Zinc (II) Phthalocyanine Polymer and Gel Formulation. Photochem. Photobiol..

[33] Saifuddin N., Raziah A.Z., Junizah A.R. (2012). Carbon Nanotubes: A Review on Structure and Their Interaction with Proteins. J. Chem..

[34] Basova T.V., Polyakov M.S. (2020). Hybrid Materials Based on Carbon Nanotubes and Polyaromatic Molecules: Methods of Functionalization and Sensor Properties. MHC.

[35] Hosu I.S., Wang Q., Vasilescu A., Peteu S.F., Raditoiu V., Railian S., Zaitsev V., Turcheniuk K., Wang Q., Li M. (2014). Cobalt Phthalocyanine Tetracarboxylic Acid Modified Reduced Graphene Oxide: A Sensitive Matrix for the Electrocatalytic Detection of Peroxynitrite and Hydrogen Peroxide. RSC Adv..

[36] Wang H., Bu Y., Dai W., Li K., Wang H., Zuo X. (2015). Well-Dispersed Cobalt Phthalocyanine Nanorods on Graphene for the Electrochemical Detection of Hydrogen Peroxide and Glucose Sensing. Sens. Actuators B Chem..

[37] Mashazi P., Mugadza T., Sosibo N., Mdluli P., Vilakazi S., Nyokong T. (2011). The Effects of Carbon Nanotubes on the Electrocatalysis of Hydrogen Peroxide by Metallo-Phthalocyanines. Talanta.

[38] Falkowski M., Rebis T., Piskorz J., Popenda L., Jurga S., Mielcarek J., Milczarek G., Goslinski T. (2016). Improved Electrocatalytic Response toward Hydrogen Peroxide Reduction of Sulfanyl Porphyrazine/Multiwalled Carbon Nanotube Hybrids Deposited on Glassy Carbon Electrodes. Dye. Pigment..

[39] Falkowski M., Kucinska M., Piskorz J., Wieczorek-Szweda E., Popenda L., Jurga S., Sikora A., Mlynarczyk D.T., Murias M., Marszall M.P. (2021). Synthesis of Sulfanyl Porphyrazines with Bulky Peripheral Substituents—Evaluation of Their Photochemical Properties and Biological Activity. J. Photochem. Photobiol. A Chem..

[40] Nar I., Atsay A., Karaoğlu H.P., Altındal A., Hamuryudan E. (2018). π-Extended Hexadeca-Substituted Cobalt Phthalocyanine as an Active Layer for Organic Field-Effect Transistors. Dalton Trans..

[41] Sevim A.M., Arıkan S., Koca A., Gül A. (2012). Synthesis and Spectroelectrochemistry of New Phthalocyanines with Ester Functionalities. Dye. Pigment..

[42] Tuncer S., Koca A., Gül A., Avcıata U. (2012). Synthesis, Characterization, Electrochemistry and Spectroelectrochemistry of Novel Soluble Porphyrazines Bearing Unsaturated Functional Groups. Dye. Pigment..

[43] Nas A., Kantekin H., Koca A. (2014). Novel 4-(2-(Benzo[d]Thiazol-2-Yl)Phenoxy) Substituted Phthalocyanine Derivatives: Synthesis, Electrochemical and in Situ Spectroelectrochemical Characterization. J. Organomet. Chem..

[44] Koca A., Gonca E., Gül A. (2008). Voltammetric and Spectroelectrochemical Characterization of Porphyrazines: Electrochemical Metal Sensor. J. Electroanal. Chem..

[45] Adebayo A.I., Nyokong T. (2009). Synthesis, Spectroscopic and Electrochemical Properties of Manganese, Nickel and Iron Octakis-(2-Diethylaminoethanethiol)-Phthalocyanine. Polyhedron.

[46] Tuncer S., Koca A., Gül A., Avcıata U. (2009). 1,4-Dithiaheterocycle-Fused Porphyrazines: Synthesis, Characterization, Voltammetric and Spectroelectrochemical Properties. Dye. Pigment..

[47] Demirbaş Ü., Kobak R.Z.U., Barut B., Bayrak R., Koca A., Kantekin H. (2016). Synthesis and Electrochemical Characterization of Tetra-(5-Chloro-2-(2,4-Dichlorophenoxy)Phenol) Substituted Ni(II), Fe(II) and Cu(II) Metallophthalocyanines. Synth. Met..

[48] Farnia G., Romanin A., Capobianco G., Torzo F. (1971). Electrochemical Reduction Mechanism of Phthalimide and Some of Its N-Derivatives in DMF. J. Electroanal. Chem. Interfacial Electrochem..

[49] Morozan A., Campidelli S., Filoramo A., Jousselme B., Palacin S. (2011). Catalytic Activity of Cobalt and Iron Phthalocyanines or Porphyrins Supported on Different Carbon Nanotubes towards Oxygen Reduction Reaction. Carbon.

[50] Coates M., Nyokong T. (2013). Characterization of Glassy Carbon Electrodes Modified with Carbon Nanotubes and Iron Phthalocyanine through Grafting and Click Chemistry. Electrochim. Acta.

[51] Wu C., Sun H., Li Y., Liu X., Du X., Wang X., Xu P. (2015). Biosensor Based on Glucose Oxidase-Nanoporous Gold Co-Catalysis for Glucose Detection. Biosens. Bioelectron..

[52] Welch C.M., Banks C.E., Simm A.O., Compton R.G. (2005). Silver Nanoparticle Assemblies Supported on Glassy-Carbon Electrodes for the Electro-Analytical Detection of Hydrogen Peroxide. Anal. Bioanal. Chem..

[53] Achraf Ben Njima M., Legrand L. (2022). Ag Nanoparticles-Oxidized Green Rust Nanohybrids for Novel and Efficient Non-Enzymatic H2O2 Electrochemical Sensor. J. Electroanal. Chem..

[54] Paulraj P., Umar A., Rajendran K., Manikandan A., Kumar R., Manikandan E., Pandian K., Mahnashi M.H., Alsaiari M.A., Ibrahim A.A. (2020). Solid-State Synthesis of Ag-Doped PANI Nanocomposites for Their End-Use as an Electrochemical Sensor for Hydrogen Peroxide and Dopamine. Electrochim. Acta.

[55] Ning R., Lu W., Zhang Y., Qin X., Luo Y., Hu J., Asiri A.M., Al-Youbi A.O., Sun X. (2012). A Novel Strategy to Synthesize Au Nanoplates and Their Application for Enzymeless H_2_O_2_ Detection. Electrochim. Acta.

[56] Bo X., Bai J., Ju J., Guo L. (2010). A Sensitive Amperometric Sensor for Hydrazine and Hydrogen Peroxide Based on Palladium Nanoparticles/Onion-like Mesoporous Carbon Vesicle. Anal. Chim. Acta.

