# Supplementary material for: A Synergistic Effect of Phthalimide-Substituted Sulfanyl Porphyrazines and Carbon Nanotubes to Improve the Electrocatalytic Detection of Hydrogen Peroxide
Source: Molecules. 2022 Jul 9;27(14):4409. doi: 10.3390/molecules27144409 (PMC9322414; doi:10.3390/molecules27144409)
Supplement: Supplementary file 1 [file molecules-27-04409-s001.zip › molecules-1786067-supplementary.pdf]

Supplementary data

for

A synergistic effect of phthalimide-substituted sulfanyl porphyrazines and carbon nanotubes to improve the electrocatalytic detection of hydrogen peroxide

Michal Falkowski[a,*], *Amanda Leda*[b], *Tomasz Rebis*[b*], *Jaroslaw Piskorz*[c], *Lukasz Popenda*[d], *Mina Hassani*[a], *Dariusz T. Mlynarczyk*[e], *Michal P. Marszall*[a], *Grzegorz Milczarek*[b]

[a] *Department of Medicinal Chemistry, Collegium Medicum in Bydgoszcz, Faculty of Pharmacy, Nicolaus Copernicus University in Torun, Dr. A. Jurasza 2, 85-089 Bydgoszcz, Poland*

[b] *Institute of Chemistry and Technical Electrochemistry, Poznan University of Technology, Berdychowo 4, 60-965 Poznan, Poland*

[c] *Chair and Department of Inorganic and Analytical Chemistry, Poznan University of Medical Sciences, Rokietnicka 3, 60-806 Poznan , Poland*

[d] *NanoBioMedical Centre, Adam Mickiewicz University in Poznan, Wszechnicy Piastowskiej 3, 61-614 Poznan, Poland*

[e] *Chair and Department of Chemical Technology of Drugs, Poznan University of Medical Sciences, Grunwaldzka 6, 60-780 Poznan, Poland*

CORRESPONDING AUTHORS*: m.falkowski@cm.umk.pl (Michal Falkowski), tomasz.rebis@put.poznan.pl (Tomasz Rebis)

Table of Contents

1. HPLC purity of porphyrazines 3 and 4..3

2. NMR study...12

Figure S1. ^1H and (^{13}C) chemical shift values [ppm] of **3** and key correlations observed in NMR spectra.
Table S1. ^1H and ^{13}C NMR data obtained for **3** including key correlations determined from ^1H-^{13}C HSQC and ^1H-^{13}C HMBC spectra.
Figure S2. ^1H NMR spectrum of **3** (800 MHz, DMSO-d_6, 298 K).
Figure S3. ^{13}C NMR spectrum of **3** (201 MHz, DMSO-d_6, 298 K).

3. Photochemical study..14

Table S2. UV–Vis absorption maxima (λ_{Abs}) and logarithms of molar absorption coefficients (logε) of Pzs **3** and **4** in selected organic solvents.

4. Electrochemical study...14

Figure S4. Cyclic voltammogram (A) and differential pulse voltammograms (B) for magnesium(II) porphyrazine **Pz1**.
Figure S5. Cyclic voltammogram (A) and differential pulse voltammograms (B) for metal-free porphyrazine **Pz2**.
Figure S6. Chronomperometric response of the GC/MWCNT/**Pz3** (A) and GC/MWCNT/**Pz4** (B)

1. HPLC purity

The purity of macrocycles **3**, **4** was determined by HPLC analysis using an Agilent 1200 instrument equipped with UV-DAD detector. The chromatographic separation was obtained on an octadecylsilane-coated column, 150 mm × 4.6 mm, 5 µm (Eclipse XDB-C18, Agilent), using gradient elution conditions at a flow rate of 1.0 mL/min. Band dispersion, and additional peaks from aggregates significantly hampered HPLC analysis. The best conditions for each compound are shown below. Peaks of minor components were detected, but the impurity content never exceeded 5% of the total signal intensity.

Porphyrazine 3

Configuration 1

	phase		
time [min]	methanol	dichloromethane	water
0	85	5	10
3	85	5	10
4	15	85	0
15	15	85	0

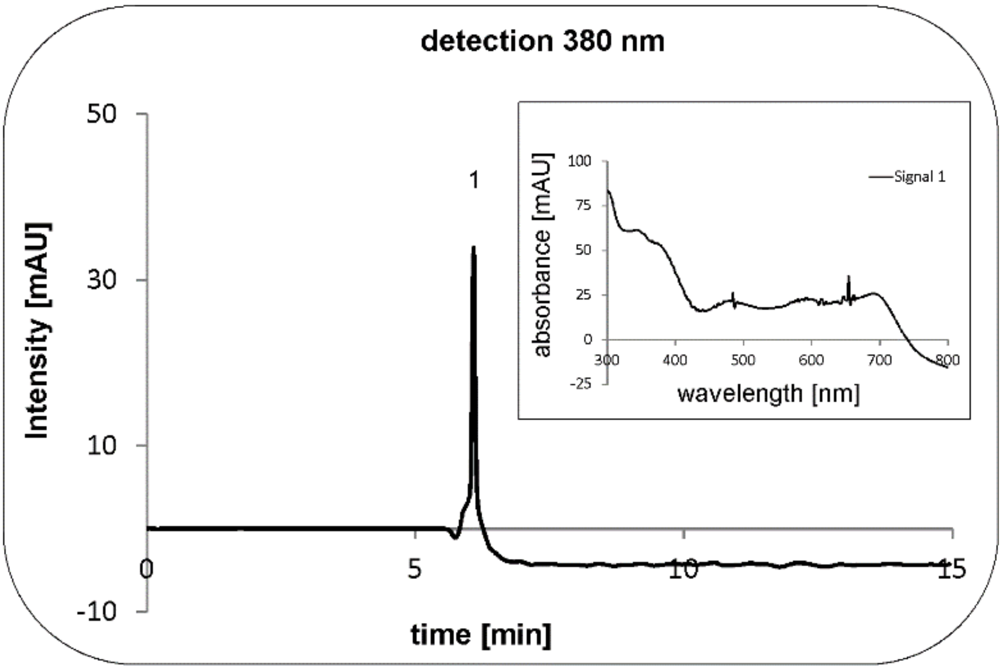

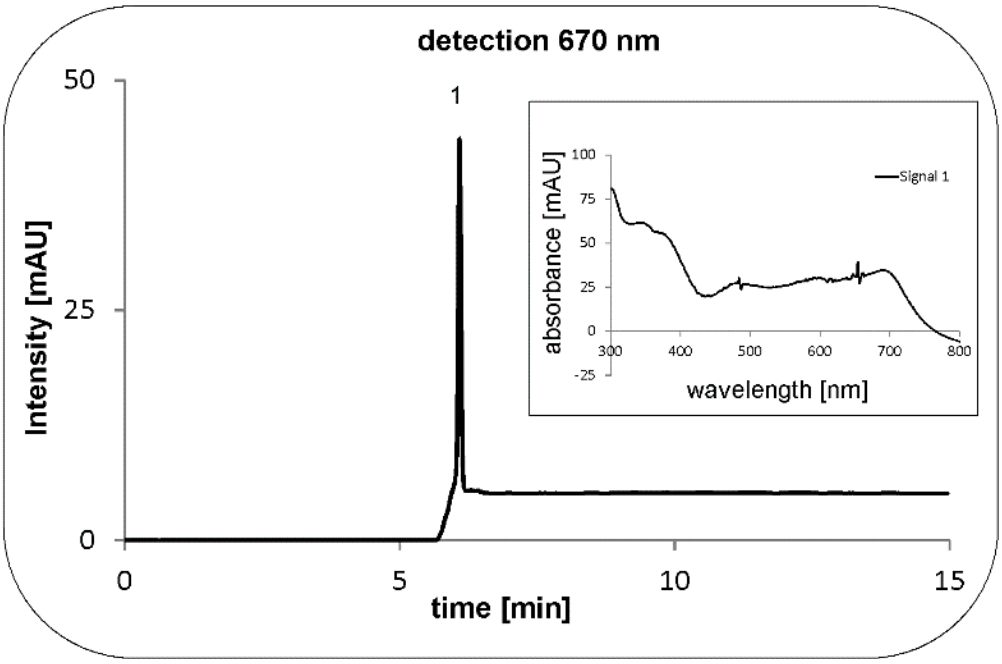

results			
signal	retention time [min]	area	purity [%]
detection 380 nm			
1	6.1	240.4	100.0
detection 670 nm			
1	6.1	199.1	100.0

Configuration 2

phase				
time [min]	methanol	acetonitrile	dichloromethane	water
0	40	40	5	15
3	40	40	5	15
4	5	5	90	0
15	5	5	90	0

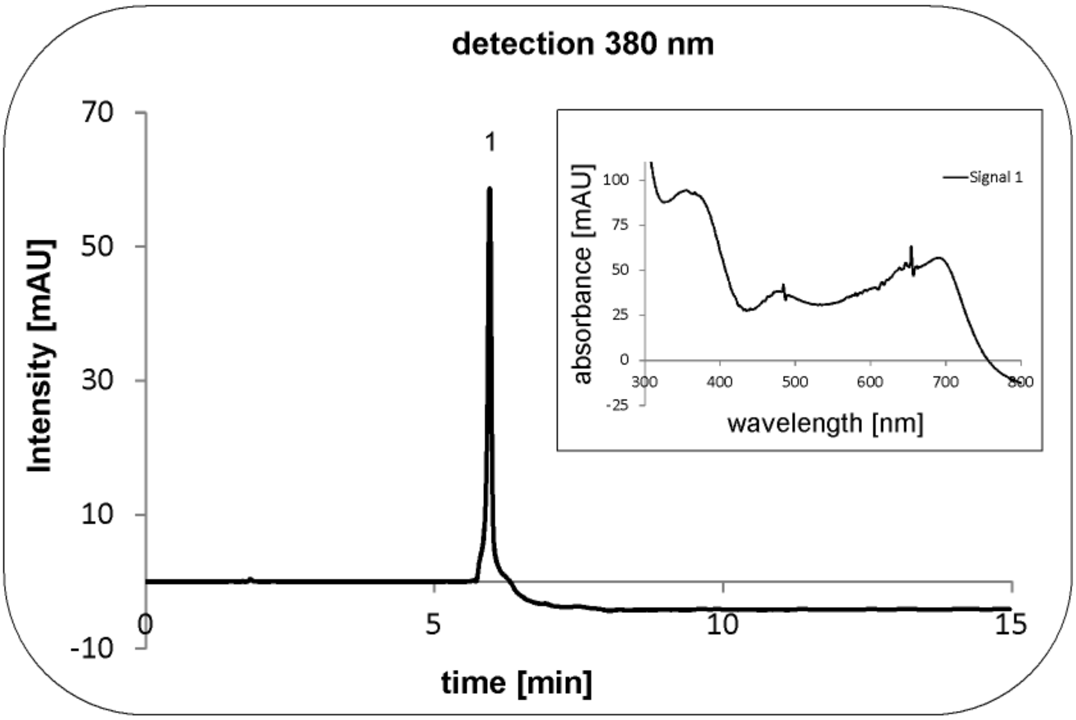

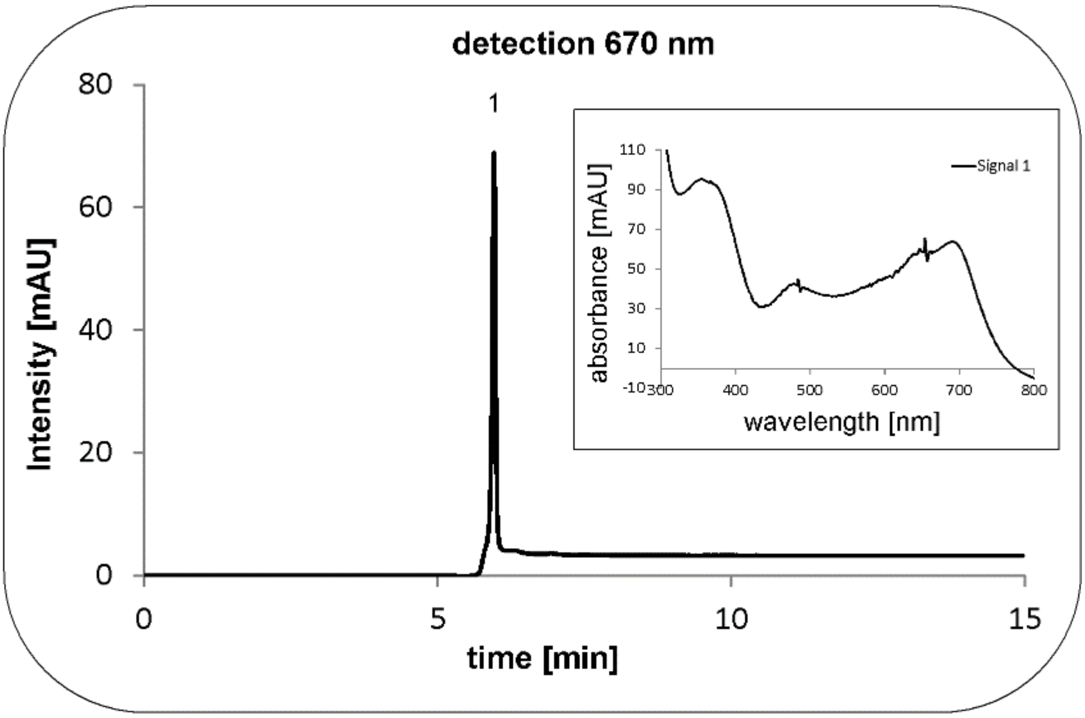

results			
signal	retention time [min]	area	purity [%]
detection 380 nm			
1	6.0	381.8	100.0
detection 670 nm			
1	6.0	320.5	100.0

Configuration 3

phase				
time [min]	methanol	acetonitrile	tetrahydrofuran	water
0	40	40	5	15
3	40	40	5	15
4	5	5	90	0
15	5	5	90	0

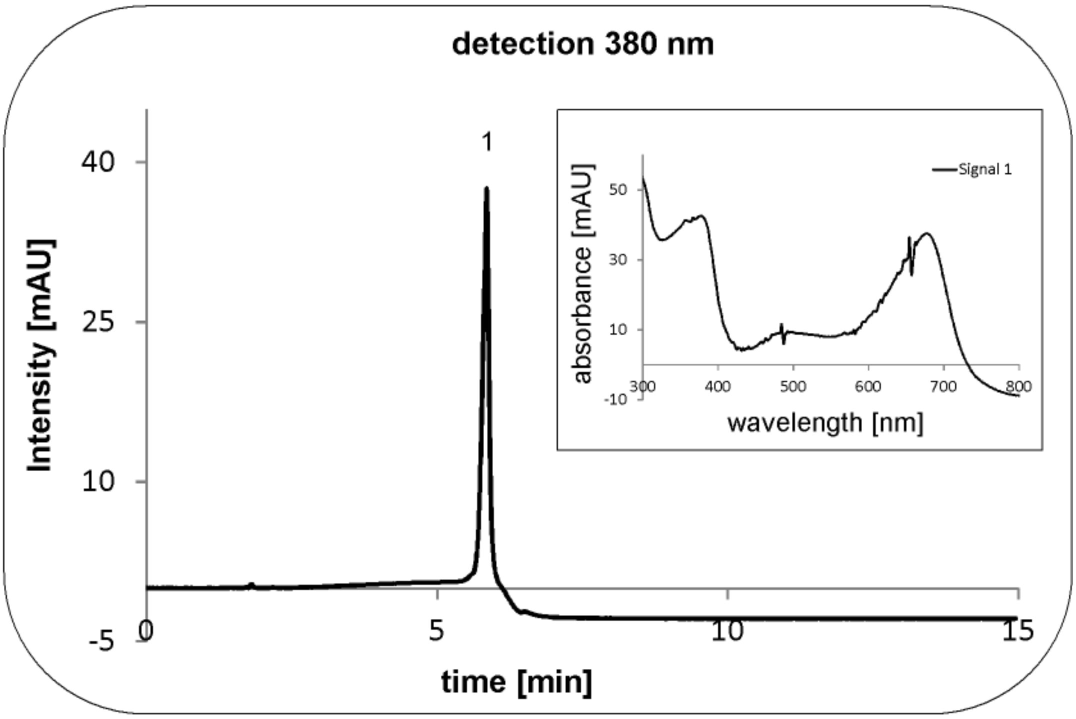

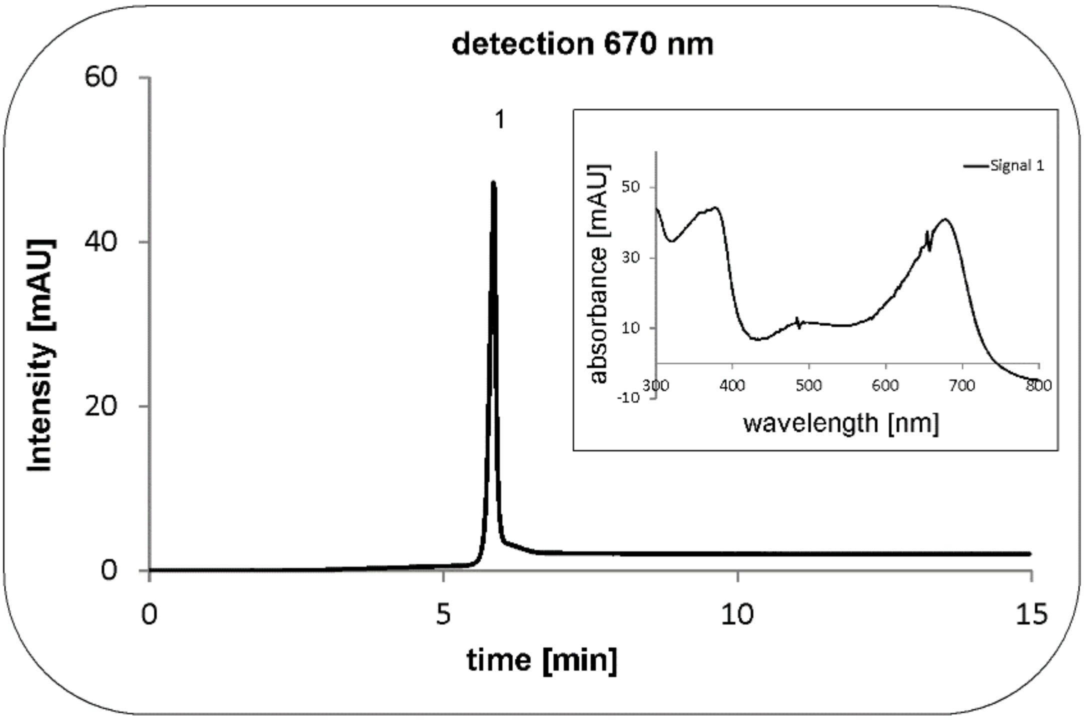

results			
signal	retention time [min]	area	purity [%]
detection 380 nm			
1	5.9	347.6	100.0
detection 670 nm			
1	5.9	357.8	100.0

Porphyrazine 4

Configuration 1

phase			
time [min]	methanol	dichloromethane	water
0	90	0	10
3	90	0	10
4	10	90	0
15	10	90	0

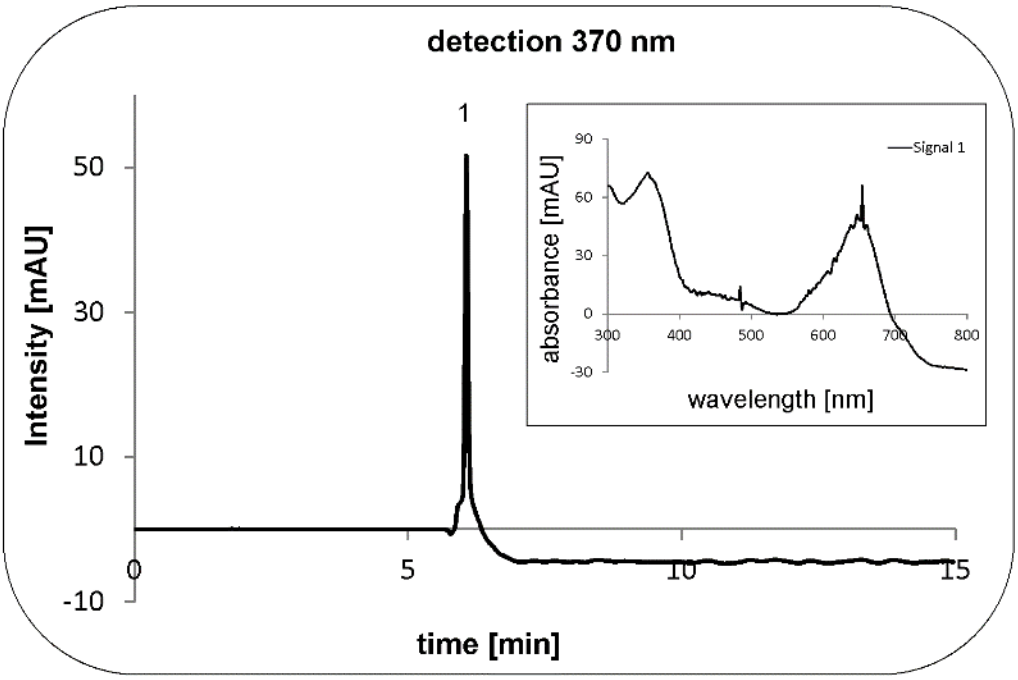

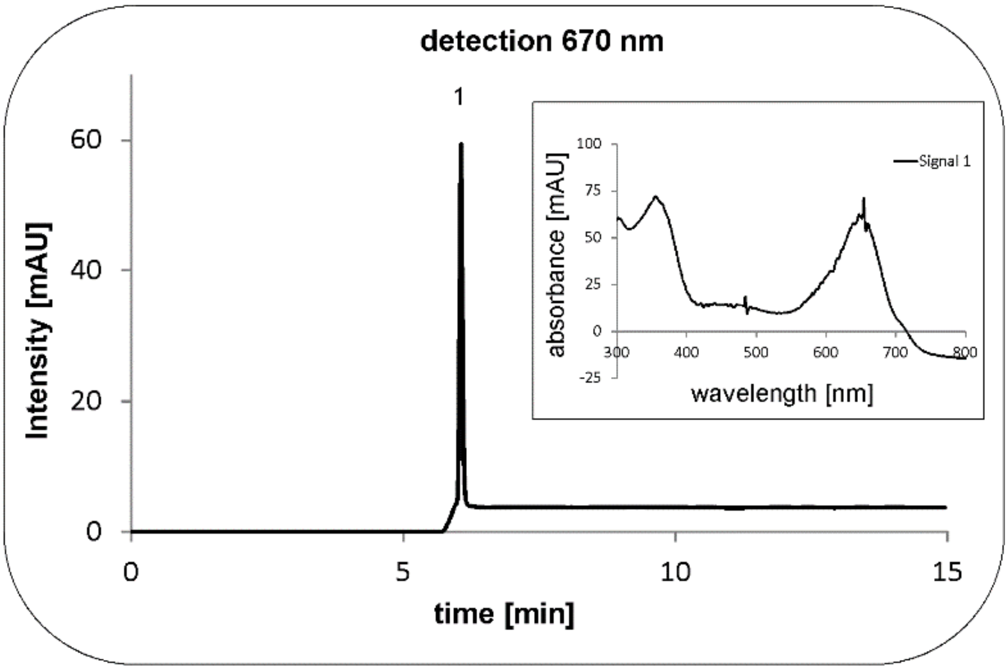

results			
signal	retention time [min]	area	purity [%]
detection 370 nm			
1	6.1	332.8	100.0
detection 670 nm			
1	6.1	247.3	100.0

Configuration 2

phase			
time [min]	methanol	tetrahydrofuran	water
0	85	0	15
3	85	0	15
4	15	85	0
15	15	85	0

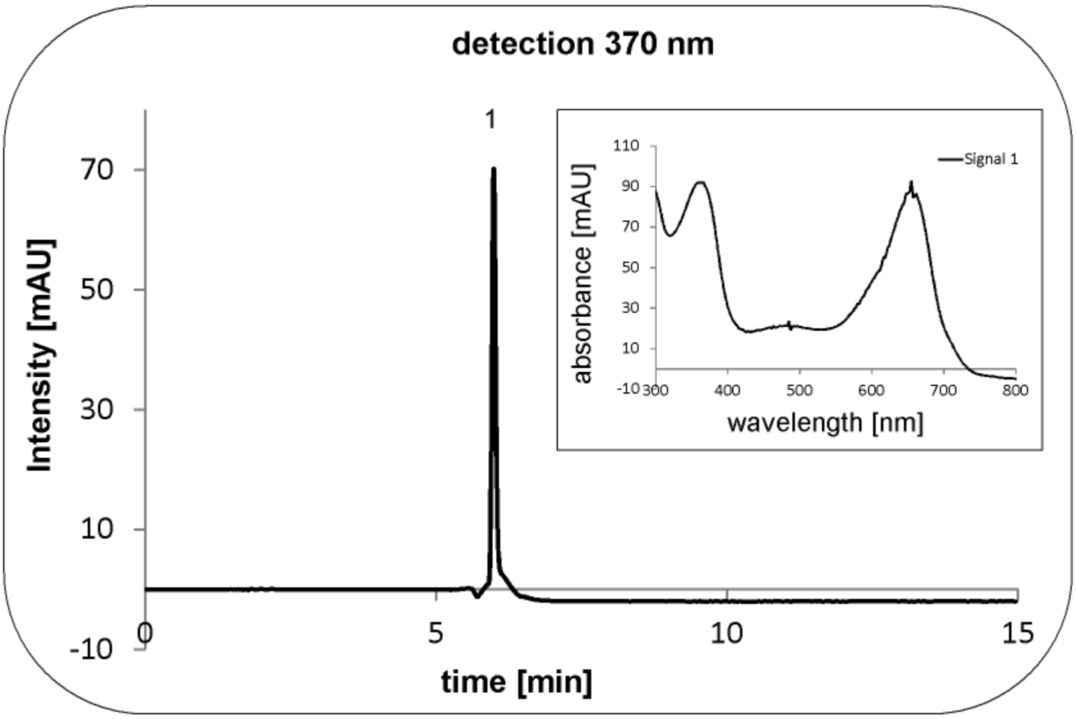

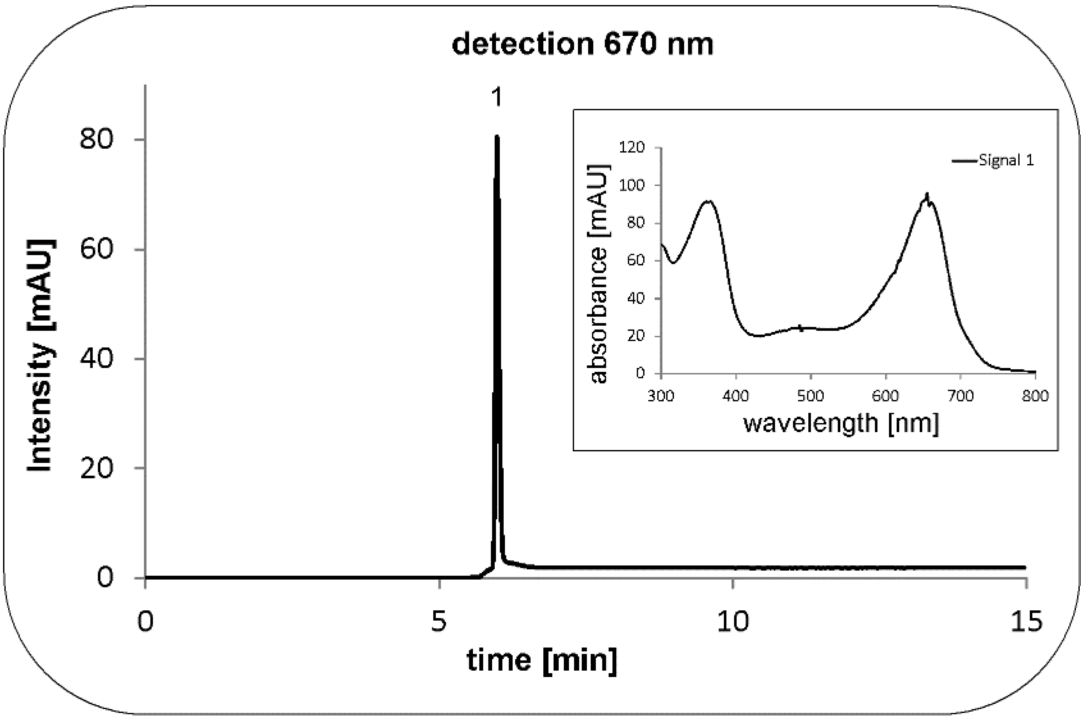

results			
signal	retention time [min]	area	purity [%]
detection 370 nm			
1	6.0	401.1	100.0
detection 670 nm			
1	6.0	363.1	100.0

Configuration 3

phase				
time [min]	methanol	acetonitrile	tetrahydrofuran	water
0	35	35	10	20
3	35	35	10	20
4	10	10	80	0
15	10	10	80	0

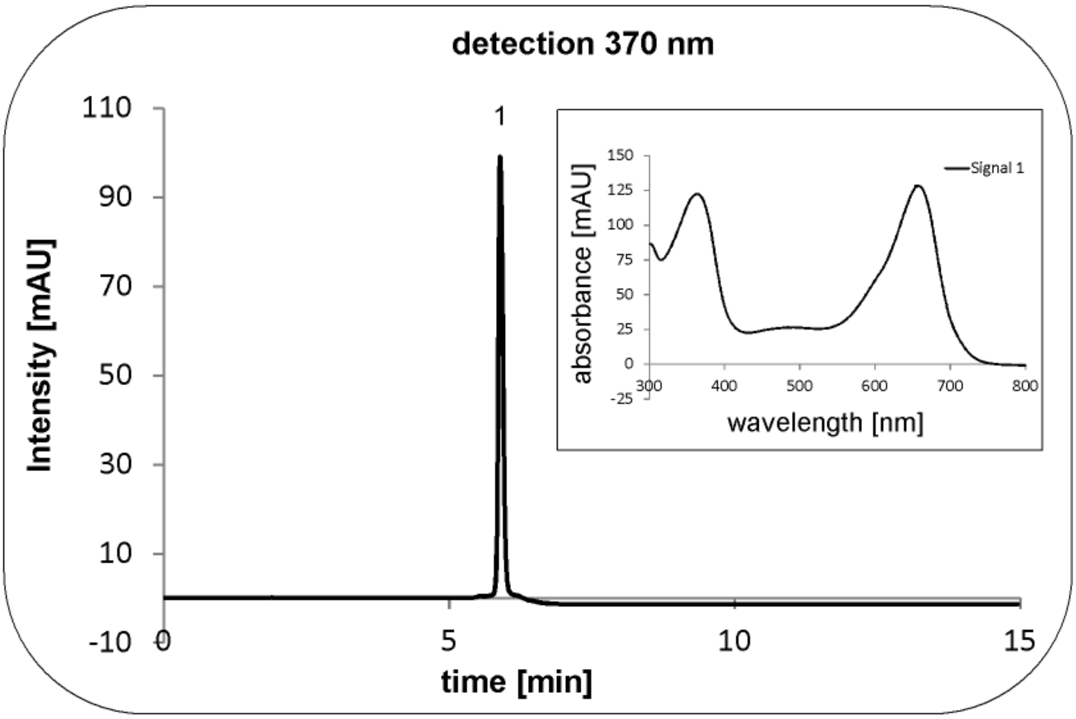

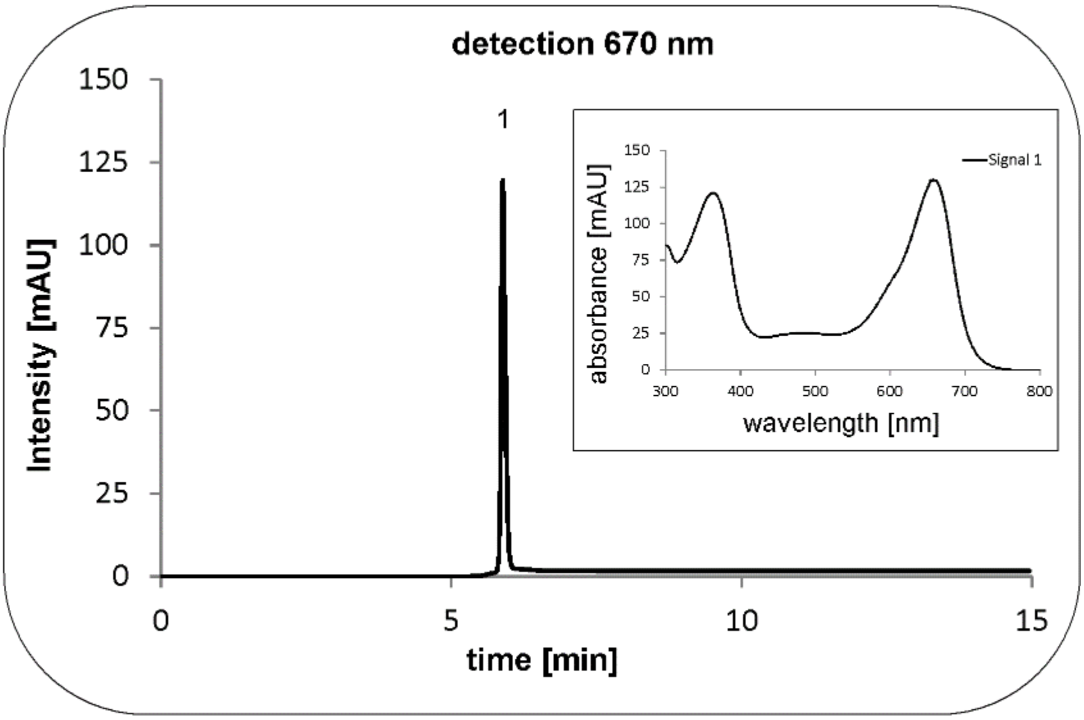

results			
signal	retention time [min]	area	purity [%]
detection 370 nm			
1	5.9	560.5	100.0
detection 670 nm			
1	5.9	609.5	100.0

2. NMR study

2.1. NMR data of 3

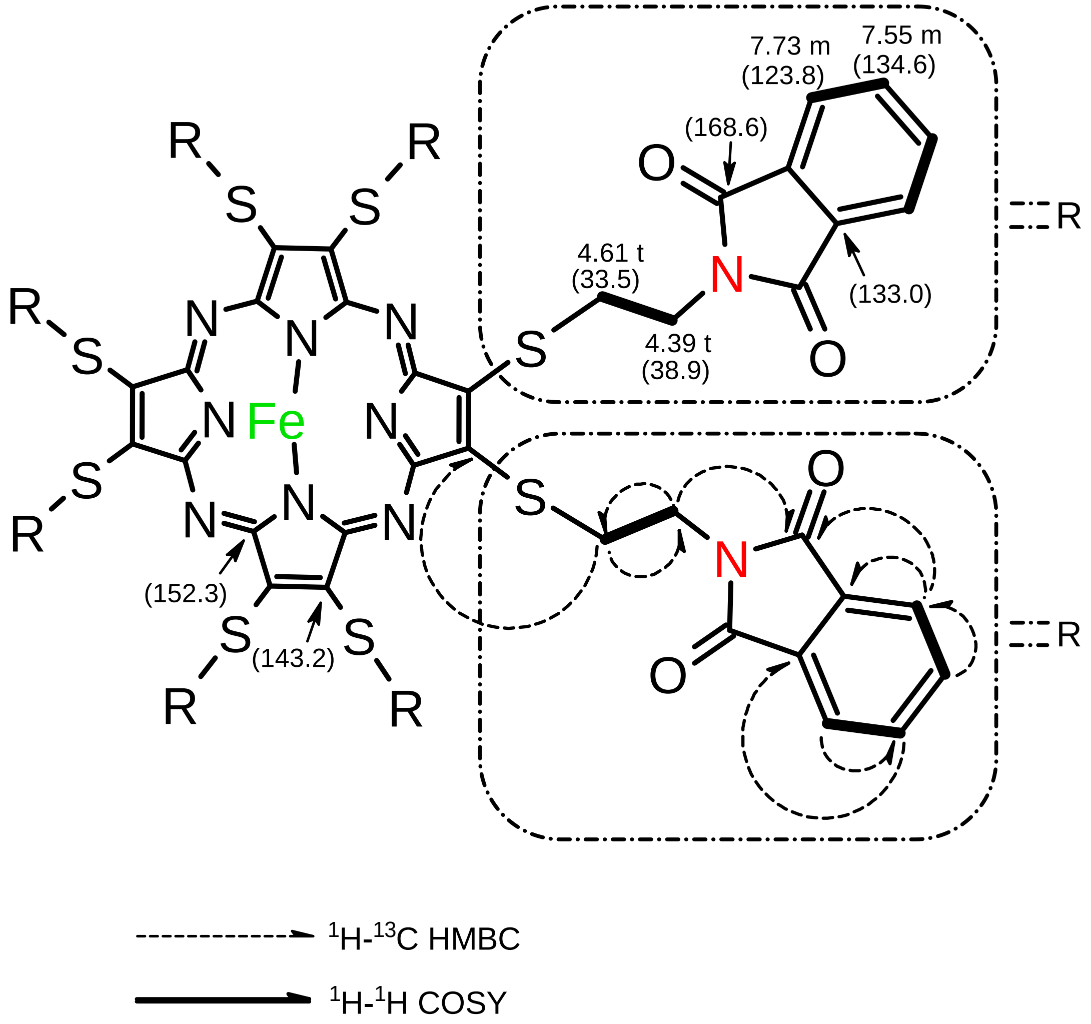

Figure S1. NMR data of **3**: ^{1}H and (^{13}C) chemical shift values [ppm] and key correlations observed in NMR spectra. Bold lines: ^{1}H-^{1}H COSY; Dashed arrows: ^{1}H-^{13}C HMBC.

Table S1. ^{1}H and ^{13}C NMR data obtained for **3** including key correlations determined from ^{1}H-^{13}C HSQC and ^{1}H-^{13}C HMBC spectra.

δ_H (ppm)	Multiplicity (J_{H-H} in Hz)	^{1}H-^{13}C HSQC δ_C (ppm)	^{1}H-^{13}C HMBC δ_C (ppm)
7.73	m	123.8	168.6 134.6 133.0
7.55	m	134.6	168.6 133.0 123.8
4.61	t (6)	33.5	143.2 38.9
4.39	t (6)	38.9	168.6 33.5
Other quaternary carbon atoms: 152.3 (ppm)			

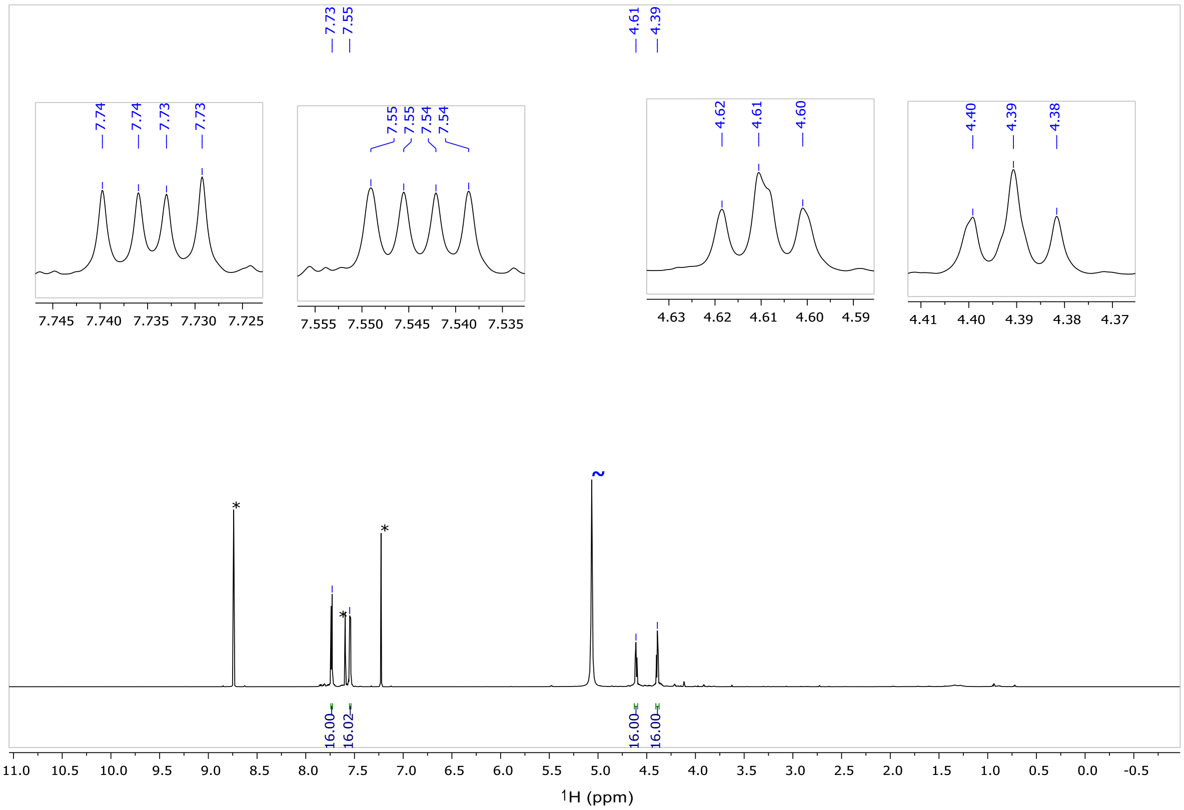

Figure S2. ^{1}H NMR spectrum of **3** (800 MHz, pyridine-d_5, 298 K). The symbols * and ~ indicate pyridine-d_5 and water residual peaks, respectively.

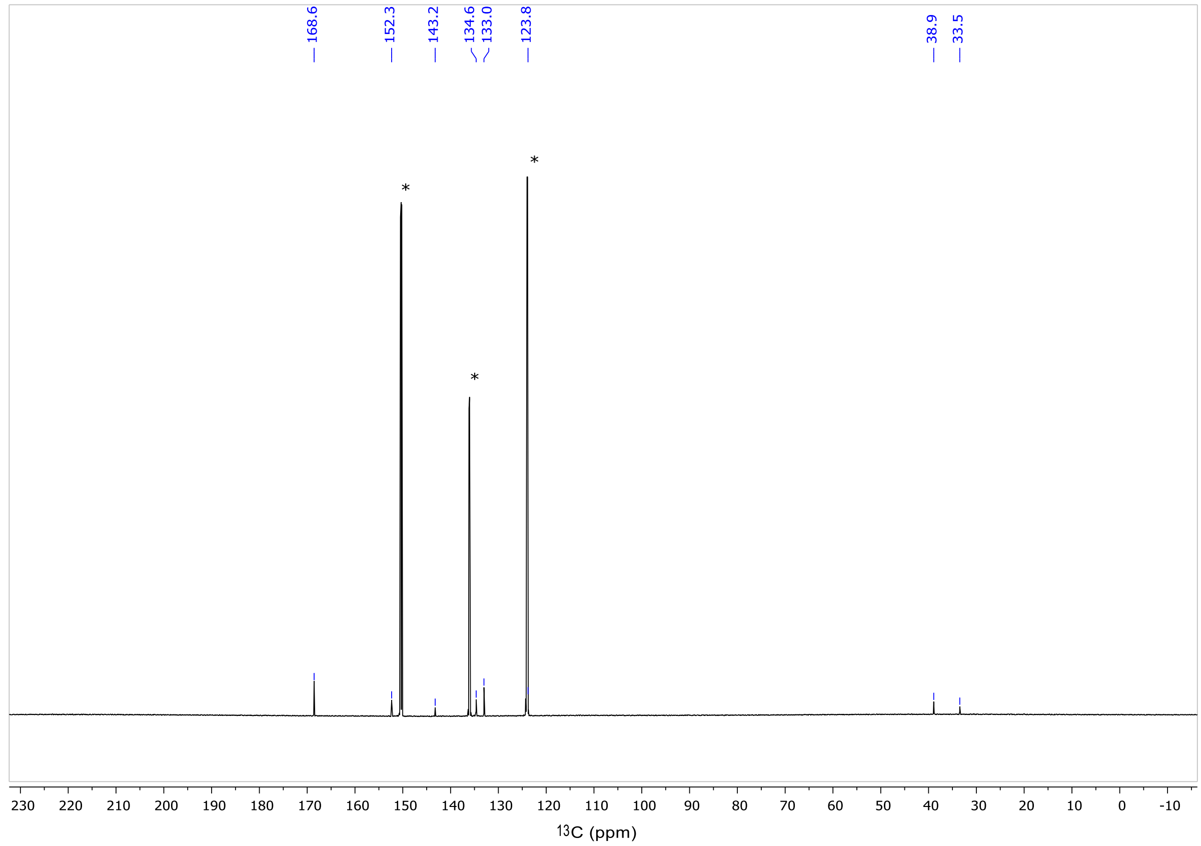

Figure S3. ^{13}C NMR spectrum recorded for **3** (201 MHz, pyridine-d_5, 298 K). The symbol * indicates pyridine-d_5 peaks.

3. Photochemical study

Table S2. UV–Vis absorption maxima (λ_{Abs}) and logarithms of molar absorption coefficients (logε) of Pzs **3** and **4** in selected organic solvents.

Solvent		Pzs			
		λ_{1Abs} (log ε)	λ_{2Abs} (log ε)	λ_{3Abs} (log ε)	λ_{4Abs} (log ε)
Dichloromethane	Pz **3**	296 (4.18)	368 (4.05)	655 (3.89)	695 (3.93)
	Pz **4**	297 (4.43)	356 (4.50)	645 (4.59)	—
N,N-Dimethylformamide	Pz **3**	291 (4.36)	365 (4.21)	648 (4.02)	692 (4.00)
	Pz **4**	300 (4.80)	358 (4.83)	646 (4.94)	—
Dimethyl sulfoxide	Pz **3**	287 (4.44)	370 (4.27)	641 (4.11)	691 (4.01)
	Pz **4**	271 (4.65)	358 (4.58)	643 (4.65)	—

4. Electrochemical study

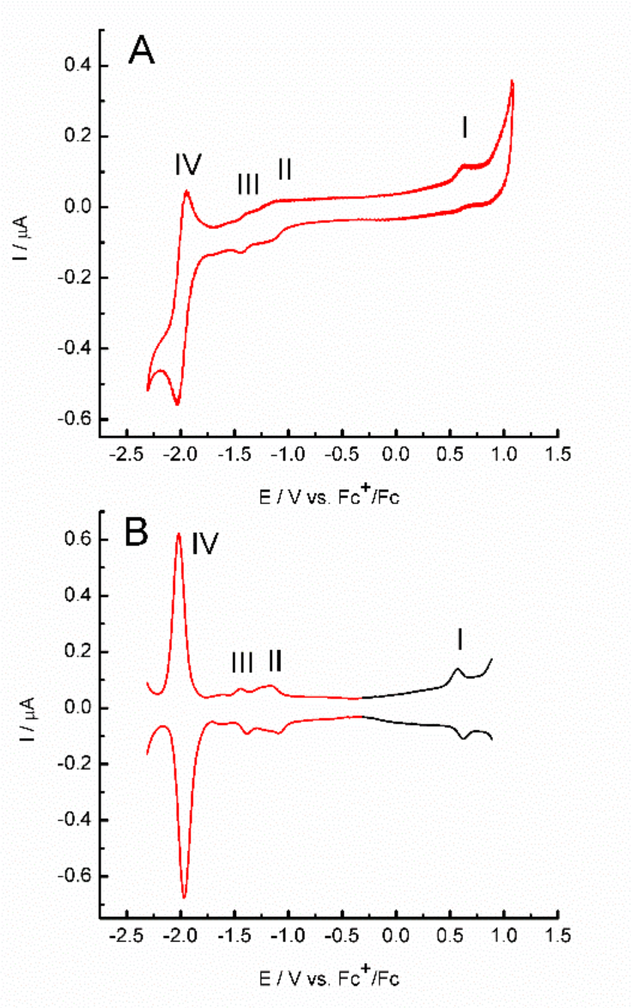

Figure S4. Cyclic voltammogram (A) and differential pulse voltammograms (B) for magnesium containing porphyrazine **Pz1**.

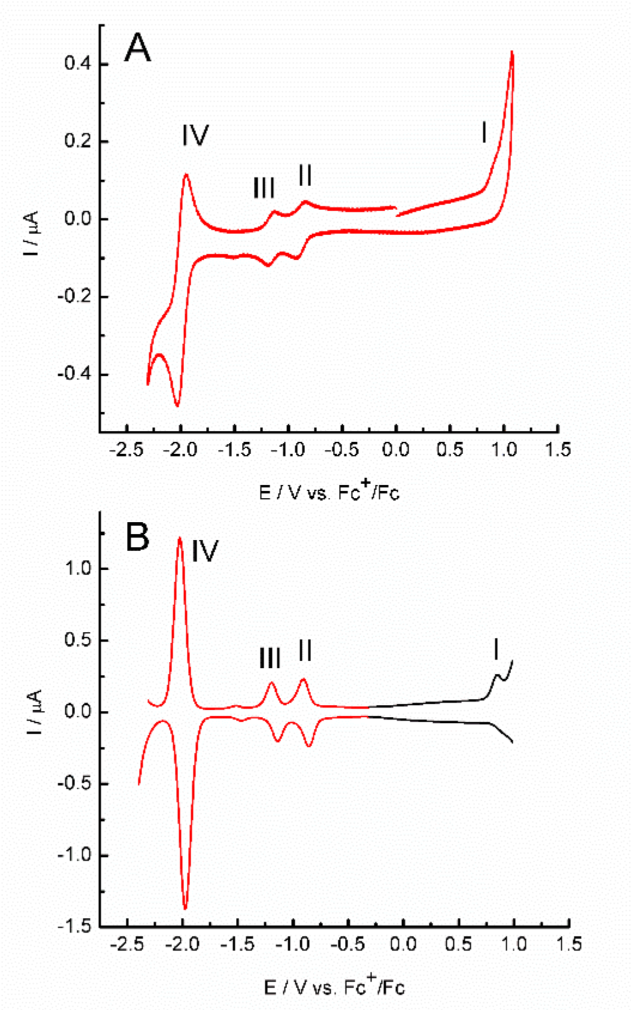

Figure S5. Cyclic voltammogram (A) and differential pulse voltammograms (B) for metal-free porphyrazine **Pz2**.

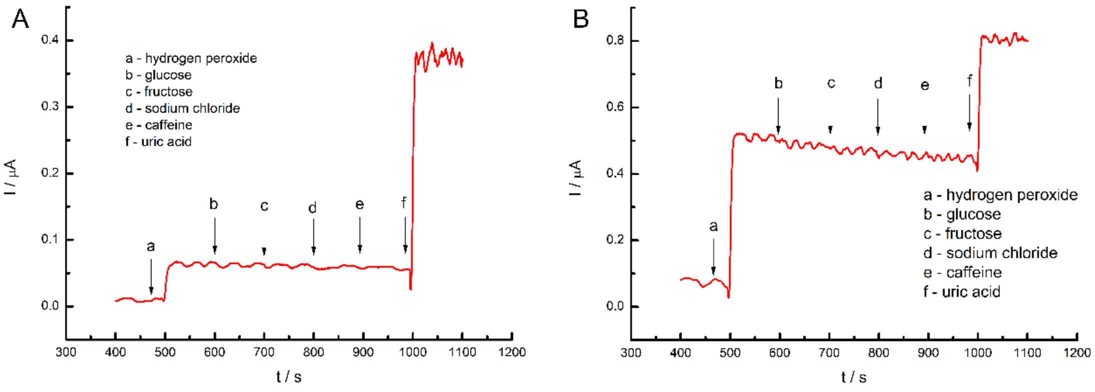

Figure S6. Chronomperometric response of the GC/MWCNT/**Pz3** (A) and GC/MWCNT/**Pz4** (B) electrodes with the addition of 50 μM H_2O_2 (a) and subsequent additions of 50 μM of interfering species in the PB solution at +0.40 V under stirring conditions.
